# Pulmonary diseases in SLE: a population-based cross-sectional study

**DOI:** 10.1136/lupus-2025-001895

**Published:** 2026-03-31

**Authors:** Henrik Zachar Langkilde, Jesper Rømhild Davidsen, Stefan Harders, Stefan Luef, Susan Due Kay, Tine Lottenburger, Stavros Chrysidis, Karen Schreiber, Elisabet Svenungsson, Sille Fløjborg, Robin Christensen, Anne Voss

**Affiliations:** 1 Research Unit of Rheumatology, Department of Clinical Research, University of Southern Denmark, Odense University Hospital, Odense, Denmark; 2 Odense Respiratory Research Unit (ODIN), Department of Clinical Research, University of Southern Denmark, Odense University Hospital, Odense, Denmark; 3 South Danish Center for Interstitial Lung Diseases (SCILS) and Pulmo-Rheuma Frontline Center (PURE) Department of Respiratory Medicine, Odense Universitetshospital, Odense, Denmark; 4 Research Unit of Department of Radiology, University of Southern Denmark, Odense Universitetshospital, Odense, Denmark; 5 Department of Rheumatology, Vejle Hospital, Vejle, Denmark; 6 Department of Rheumatology, University Hospital of Southern Denmark, Esbjerg and Grindsted Hospital, Esbjerg, Denmark; 7 Danish Center for Expertise in Rheumatology, Danish Hospital for Rheumatic Diseases, Sønderborg, Denmark; 8 Institute for Regional Health Research, Southern Danish University, Sønderborg, Denmark; 9 Medicine Solna, Unit of Rheumatology, Karolinska Institutet/Karolinska University Hospital, Stockholm, Sweden; 10 Systemic Lupus Erythematosus Patient Research Partner, Department of Rheumatology, Odense Universitetshospital, Odense, Denmark; 11 Section for Biostatistics and Evidence-Based Research, Parker Instituttet, Copenhagen, Denmark; 12 Department of Clinical Research, University of Southern Denmark Faculty of Health Sciences, Odense, Denmark

**Keywords:** Systemic Lupus Erythematosus, Pulmonary Fibrosis, Prevalence

## Abstract

**Objectives:**

Pulmonary diseases (PDs) in SLE are thought to be common and of clinical importance but can be difficult to diagnose. Prior studies report divergent estimates of the prevalence of PD in SLE ranging from 30% to 98%. Therefore, our primary objective was to determine the prevalence of PD in patients with SLE, through a population-based cross-sectional study. Our secondary objectives were to investigate in patients with SLE: (1) the prevalence of specific subtypes of PD, (2) pulmonary function and (3) associations between selected patient characteristics and presence of PD and subtypes of PD.

**Methods:**

We invited adult patients with SLE who met the classification criteria and lived in Region Southern Denmark. Each participant underwent a clinical assessment, pulmonary function tests (PFTs) and a chest high-resolution CT scan (HRCT). Subsequently, participants were assessed and diagnosed according to the presence of PD and subtypes of PD at a multidisciplinary discussion.

**Results:**

We found PDs were prevalent in 109 (59%) of the 185 included participants, among whom interstitial lung diseases were prevalent in 22 (12%), pleural diseases in 35 (19%) and airway diseases in 70 (38%). PD and subtypes of PD were associated with decreased pulmonary function, but the PFT measures showed a low combined sensitivity and specificity for detecting PD and subtypes of PD. We found no strong associations between selected patient characteristics and the presence of PD or subtypes of PD.

**Conclusion:**

59% of patients with SLE have PD, and PD was associated with decreased pulmonary function. Diagnosing PD in SLE remains challenging because clinical assessment and PFTs have limited utility. Accurate diagnosis often requires HRCT or other specialised investigations.

**Trial registration number:**

NCT06087523.

**WHAT IS ALREADY KNOWN ON THIS TOPIC**

- Pulmonary diseases (PDs) are estimated to affect 50% of patients with SLE, but the numbers are diverging between studies. PD in SLE is associated with worse outcomes such as increased mortality and decreased quality of life.

**WHAT THIS STUDY ADDS**

- This is the first population-based study that investigates PD in SLE by dedicated investigations including chest high-resolution CT scans. The study provides novel insights into the prevalence of PD in SLE, its clinical consequences and diagnostic approaches to PD.

**HOW THIS STUDY MIGHT AFFECT RESEARCH, PRACTICE OR POLICY**

- We found that PD affects 59% of patients with SLE, and PD includes 12% interstitial lung diseases, 19% pleural diseases and 38% airway diseases. We show that PD in SLE is difficult to diagnose based on regular information from outpatient clinic and pulmonary function tests (PFTs). PD in SLE seems important as they are associated with decreased PFT measures, breathlessness and cover diseases associated with an increased risk of infection.

## Introduction

 SLE is an autoimmune disease, defined by the presence of specific autoantibodies and characteristic involvement of multiple organs.^1^ Pulmonary diseases (PDs) in SLE are common and cover several types of PD including interstitial lung diseases (ILDs), pleural diseases, airway diseases and shrinking lung syndrome (SLS).^2 3^ Symptoms of PD in SLE range from severe to vague,^3^ sometimes indistinguishable from symptoms of musculoskeletal and cardiovascular origin. Additionally, a poor association has been noted between how patients and physicians report pulmonary symptoms in SLE.^4^ PD, especially ILD, in SLE is associated with higher mortality^5^ and a decreased health-related quality of life (HRQL).^6^

The prevalence of PD in SLE varies depending on the study design and definition of PD. For instance, the prevalence is 30% PD in SLE in a register-based study^5^ and 98% in an autopsy study.^7^ One study investigating SLE-related ILD (SLE-ILD) found that 4% of the study participants had SLE-ILD according to registered diagnoses, and 16% were diagnosed with ILD after dedicated clinical investigations.^8^ Studies concerning PD in SLE often focused on specific types of PD, such as ILD,^8^ and other studies primarily reported the prevalence of abnormalities in specific investigations, for example, abnormal pulmonary function tests (PFTs).^9^

Because prior studies have reported divergent findings regarding PD in SLE, we conducted this population-based, cross-sectional study to determine its prevalence of PD among Danish patients with SLE. The secondary objectives were to investigate: (1) the prevalence of specific PD subtypes, (2) PFT results, (3) associations between specific patient characteristics and the presence of PD and its subtypes.

## Materials and methods

### Study design and setting

We conducted a population-based, cross-sectional study including adult patients with SLE residing in the Region of Southern Denmark. Each participant attended a single study visit that included a clinical examination, PFT and chest high-resolution CT scan (HRCT). The presence of PD and its subtypes was determined at a multidisciplinary discussion (MDD). Data were managed in Research Electronic Data Capture (REDCap), and the study is reported in accordance with the Strengthening the Reporting of Observational Studies in Epidemiology guideline^10^ (online supplemental material 1). In the present study, the term PD covers diseases of the lung parenchyma, pleura, airways, diaphragm and pulmonary vasculature.

### Participants, ethics and registration

Patients were identified through diagnosis registries followed by a systematic search of hospital records across all institutions in the Region of Southern Denmark for visits between 1 July 2021 and 30 June 2023, with codes related to SLE or SLE-associated diseases (International Classification of Diseases, 10th Revision codes DL184A, DL930, DL930A, DM321, DM328 and DM329). In Denmark, patients with SLE are followed at public hospitals at least yearly, and all visits are registered with diagnosis codes. Possible participants were identified, their medical records were reviewed and they were invited if they met the eligibility criteria. If there was doubt of eligibility, the patient’s treating physician was consulted. Prior to the first participant’s inclusion, the study was registered at ClinicalTrials.gov (NCT06087523). The study protocol, the statistical analyses plan (SAP) and final manuscript were reviewed by a patient partner. After informed consent, participants were enrolled between 1 August 2023 and 31 October 2024. Inclusion criteria were: Diagnosis of SLE according to Fries and Holman principle,^11^ fulfilment of 2019 European League Against Rheumatism/American College of Rheumatology Classification Criteria for SLE (EULAR/ACR 2019),^12^ age ≥18 years, residence in the Region Southern Denmark and no linguistic barriers. Exclusion criteria were: Not having native lungs. We reviewed medical records and interviewed participants focusing on pulmonary symptoms including prior pleuritic pain, SLS and asthma.

### Questionnaires

Participants filled in two questionnaires in REDCap: A non-validated Danish questionnaire inspired by the Lupus Impact Tracker,^13^ and a validated Danish version of Systemic Lupus Activity Questionnaire.^14^ Participants were questioned concerning ‘dry cough’ and ‘tightness in their chest’ inspired by Liu *et al*.^15^

### Clinical evaluation

SLE disease activity was scored according to SLE Disease Activity Index 2000 (SLEDAI-2K)^16^ and Physicians Global Assessment of activity.^17^ Fulfilment of the Definition of Remission in Systemic Lupus Erythematosus^18^ and Lupus Low Disease Activity State^19^ was registered. SLE-related damage was registered according to Systemic Lupus International Collaborating Clinics/ACR Damage Index (SDI),^20^ and the SDI without contribution from PD (SDI no-PD) was noted.

### High-resolution CT scans

Participants underwent a HRCT on the visit day (online supplemental material 2 that contains details concerning the scan). If participants had a HRCT or a CT scan (CT) of sufficient quality, according to an experienced thoracic radiologist (SH), performed within the prior 6 months, it replaced the study HRCT. The scans were evaluated by an experienced thoracic radiologist (SH) blinded to other information.

### Pulmonary function tests

Participants performed PFT according to guidelines^21^,^24^ guided by a specialist nurse experienced in pulmonary medicine, registering forced expiratory volume in 1 s (FEV_1_), forced vital capacity (FVC), the FEV_1_/FVC ratio, total lung capacity (TLC), diffusing capacity for the lung for carbon monoxide (DLCO) and carbon monoxide transfer coefficient. From the 6-minute walk test (6MWT), the nurse registered the walking distance and oxygen saturation. Breathlessness was assessed using the Medical Research Council Dyspnoea Scale (MRC).^25^ Results were compared with age-specific, height-specific, ethnicity-specific and sex-specific reference values. According to current practice, PFT measures <80% of reference value were considered abnormal, except FEV1/FVC ratio (threshold 70%).

### Blood and urine samples

Blood and urine samples were collected on the visit day and analysed to calculate SLEDAI-2K and SDI.

### Classifying diagnoses of pulmonary diseases

Participants were diagnosed and classified according to subtype of PD at a MDD, according to reference standard,^26^ involving two experienced pulmonologists (JRD and SL), an experienced thoracic radiologist (SH) and an experienced rheumatologist with special interest in SLE (AV).

### Statistical methods

Prior to the unblinding of data, we developed an SAP, available online at zeonodo.org, as encouraged to enhance transparency.^27^ In online supplemental material 3, the SAP and its deviations SAP are presented.

Categorical variables are presented as frequencies and percentages, while continuous data are presented as means±SD if approximately normally distributed and otherwise as medians with IQRs. Means for different strata were compared including the difference with 95% CIs. 95% CI were calculated log transformed and then exponentiated before reporting. Medians were compared pairwise with quantile regression noting differences and 95% CI. Differences between two prevalences were compared noting risk differences. Groups in different strata were compared with standardised differences (StdD), values <0.1 indicate negligible differences and values >0.2 suggest meaningful imbalance.

By univariable logistic regression, we calculated ORs for associations between the outcome PD or its subtypes and certain predefined variables; subsequently, statistically significant associations were investigated with multivariable regression analyses. Post hoc, we developed graphs of cumulative incidence proportions, fitted multivariable regression analyses by Wald test and eliminating collinear variables and an overview of diagnostic characteristics of PFT. All analyses were performed in Excel or StataBE V.18.0 (64-bit).

## Results

### Study participants

**Figure 1:**
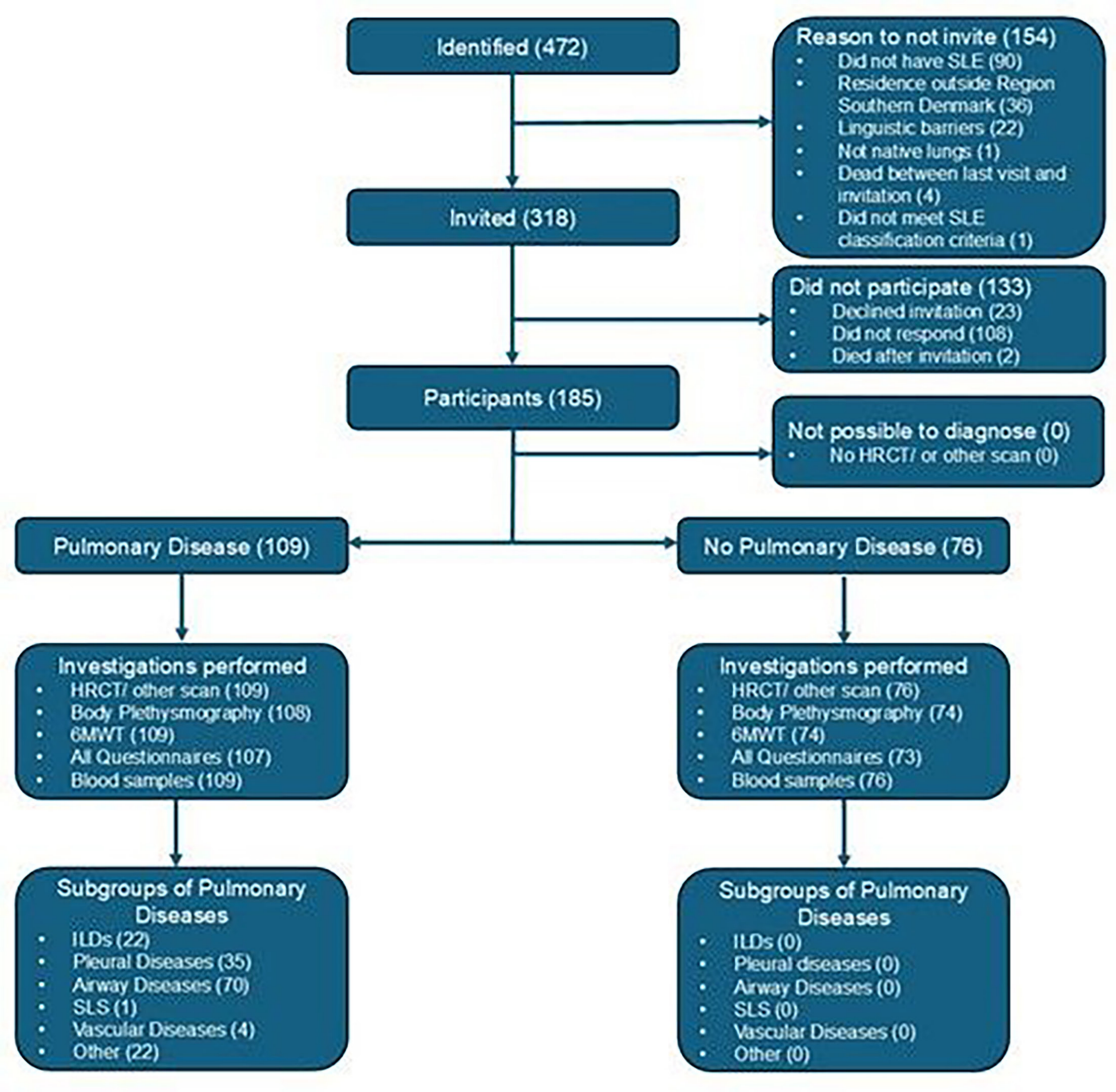
Flow chart illustrating the identification, invitation, inclusion, participation in investigations and diagnoses of participants in the study. HRCT, high-resolution CT scan; ILD, interstitial lung disease; 6MWT, 6-minute walk test; SLS, shrinking lung syndrome.

We invited 318 patients, and 185 (58%) participated (figure 1). Participants had a mean age of 52 years; 160 (87%) were female; the median disease duration was 15 years; most were white (94%); and 54% had ever smoked. Our primary search identified 472 possible participants; 154 were not invited, mostly because they did not have SLE or lived outside the Region Southern Denmark. Based on available data for non-participants (n=133), their mean age was 53 years (StdD 0.093) and 121 (91%) were female (StdD 0.142).

Participants had a median SLEDAI-2K score of 4 and participants with PD had a lower disease activity than those without PD. Participants had a median SDI of 1, participants with PD had a higher median SDI than those without PD (StdD=0.713, but no difference in SDI no-PD (online supplemental Appendix 1). Most had been treated with corticosteroid, hydroxychloroquine and conventional disease modifying antirheumatic drugs, while 36 (19%) had received biologic disease modifying antirheumatic drugs. Almost half had other overlapping autoimmune rheumatic diseases (ARDs) and/or secondary ARD, most commonly antiphospholipid syndrome (APS). Except for age, there were only minor differences between participants with and without PD (table 1). PD did not relate to self-reported HRQL or SLE disease activity (online supplemental Appendix 1). Participants with PD had a higher median MRC, and PD was associated with abnormal PFT measures. However, no 6MWT measures were associated with PD except decreased saturation and pleural diseases. Among participants without PD, 46% have abnormally low DLCO (online supplemental Appendix 2a). The subtypes of PD were associated with decreased PFT measures (online supplemental Appendix 2b–d).

**Table 1:** Baseline characteristics of 185 patients with SLE, stratified according to presence and absence of PDs

Baseline characteristics	All	PD Present	PD Absent	Std. difference*
Age, years	52.0±14.3	55.7±14.3	46.3±12.5	0.693
Disease duration, years†	15.0 (6.51–24.5)	16.1 (7.93–27.8)	12.9 (4.62–19.9)	0.316
Female sex, n (%)	160 (87)	91 (84)	69 (91)	0.219
White population, n (%)	173 (94)	104 (95)	69 (91)	0.183
*SLE Classification criteria*				
EULAR/ACR 2019, score 0–51^12^	27.1±7.58	28.1±7.67	25.7±7.28	0.321
SLICC 2012, score 0–17†^28^	8 (7–10)	8 (7–10)	8 (7–10)	0.234
*Other SLE scores*				
SLEDAI 2K, score 0–105†^16^	4 (2–6)	2 (0–5)	4 (2–6)	0.274
SDI, score 0–48†^20^	1 (0–3)	2 (1–4)	1 (0–2)	0.713
SLAQ, score 0–100†^50^	15 (9–22)	16 (10–22)	14 (9–24)	0.017
*Biomarkers*				
anti-dsDNA positive ever, n (%)	165 (89)	98 (90)	67 (88)	0.056
aPL positive ever, n (%)	86 (46)	59 (54)	27 (36)	0.381
Decreased complement ever, n (%)	138 (75)	81 (74)	57 (75)	0.016
*Others*				
Smoking, status				0.336‡
Never, n (%)	86 (46)	47 (43)	39 (51)	0.165
Prior, n (%)	80 (43)	52 (48)	28 (37)	0.221
Present, n (%)	19 (10)	10 (9.2)	9 (12)	0.087
BMI, kg/m^2^	25.5 (23.0–28.9)	25.5 (23.0–29.0)	25.5 (22.9–28.5)	0.070
Overlap to other ARD, all, n (%)	88 (48)	56 (51)	32 (42)	0.187
Antiphospholipid syndrome, n (%)	42 (23)	30 (28)	12 (16)	0.288
Sjögren’s disease, n (%)	25 (14)	15 (14)	10 (13)	0.018
Rheumatoid arthritis, n (%)	18 (10)	12 (11)	6 (7.9)	0.107
Systemic sclerosis, n (%)	3 (1.6)	1 (0.92)	2 (2.6)	0.130
Prior mixed connective tissue disease, n (%)	10 (5.4)	6 (5.5)	4 (5.3)	0.011
*Treatment*				
Corticosteroid ever, n (%)	167 (90)	102 (94)	65 (86)	0.266
Corticosteroid current, n. (%)	49 (26)	33 (30)	16 (21)	0.212
Hydroxychloroquine ever, n (%)	185 (100)	109 (100)	76 (100)	0.000
Hydroxychloroquine current, n (%)	143 (77)	80 (73)	63 (83)	0.231
Other csDMARDs ever, n (%)	154 (83)	94 (86)	60 (79)	0.193
Other csDMARDs current, n (%)	83 (45)	49 (45)	34 (45)	0.004
bDMARDs ever, n (%)	36 (19)	20 (18)	16 (21)	0.068
bDMARDs current, n (%)	17 (9.2)	9 (8.3)	8 (11)	0.078

Values are expressed in means±SDs unless otherwise indicated.

* The reported standardised difference is the difference of the reported prevalence for each variable between patients with and without PD.

† Median and IQR.

‡ P value.

anti-dsDNA, anti-double stranded DNA antibodies; aPL, antiphospholipid antibodies; ARD, autoimmune rheumatic disease; bDMARDs, biologic disease-modifying antirheumatic drugs; BMI, body mass index; csDMARDs, conventional disease-modifying antirheumatic drugs; EULAR/ACR 2019, 2019 European League Against Rheumatism/American College of Rheumatology Classification Criteria for SLE; PD, pulmonary disease; SDI, Systemic Lupus International Collaborating Clinics/American College of Rheumatology Damage Index for SLE; SLEDAI 2K, SLE Disease Activity Index 2000; SLICC, Systemic Lupus International Collaborating Clinics.

### Prevalence of pulmonary diseases

Based on MDD 109 (59%) of the participants were diagnosed with any PD and 22 (12%) with ILD (table 2). The most common radiological ILD pattern observed was usual interstitial pneumonia, but other types included fibrotic non-specific interstitial pneumonia and lymphocytic interstitial pneumonia (online supplemental Appendix 3). Pleural diseases were found in 35 (19%). Airway diseases were the most frequent subtype of PD affecting 70 (38%) and included bronchiectasis and emphysema. One participant had SLS.

**Table 2:** Prevalence of PDs among 185 patients with SLE

Types of PD	Diagnoses of PD	Prevalence, no. (%)	95% CI
Any PD		109 (59)	52 to 66
ILD		22 (12)	7.9 to 17
	UIP	1 (0.54)	0.08 to 3.8
	Probable UIP	4 (2.2)	0.8 to 5.7
	Indeterminate for UIP	5 (2.7)	1.1 to 6.4
	Fibrotic NSIP	1 (0.54)	0.08 to 3.8
	Non-fibrotic NSIP	0	0
	Fibrotic HP	2 (1.1)	0.3 to 4.3
	Non-fibrotic HP	1 (0.54)	0.08 to 3.8
	Pneumonitis	0	0
	Diffuse alveolar haemorrhage	0	0
	LIP	3 (1.6)	0.5 to 4.9
	OP	1 (0.54)	0.08 to 3.8
	Other*	6 (3.2)	1.5 to 7.1
Pleural diseases		35 (19)	14 to 25
	Pleuritis	9 (4.9)	2.5 to 9.1
	Pleural disease of diaphragm	1 (0.54)	0.08 to 3.8
	Sequelae of pleuritis	23 (12)	8.4 to 18
	Other*	3 (1.6)	0.5 to 4.9
Airway diseases		70 (38)	31 to 45
	Asthma	1 (0.54)	0.08 to 3.8
	Bronchiectasis	28 (15)	11 to 21
	Obliterative bronchiolitis	3 (1.6)	0.5 to 4.9
	Infectious bronchiolitis	0	0
	COPD	8 (4.3)	2.2 to 8.4
	Emphysema	20 (11)	7.1 to 16
	Thickened airways	25 (14)	9.3 to 19
	Other*	5 (2.7)	1.1 to 6.4
SLS		1 (0.54)	0.08 to 3.8
Vascular		6 (3.2)	1.5 to 7.1
	Pulmonary embolism	0	0
	Pulmonary arterial hypertension†	5 (2.7)	1.1 to 6.4
	Pulmonary vasculitis	0	0
	Other*	1 (0.54)	0.08 to 3.8
Other*		17 (9.2)	5.8 to 14

The percentage is of the total study population. The 95% CI is calculated with log transformation and then exponentiated back.

* See online supplemental material 4.

† Evaluated on HRCT, not confirmed by heart catheterisation.

COPD, chronic obstructive pulmonary disease; HP, hypersensitivity pneumonitis; HRCT, high-resolution CT scan; ILD, interstitial lung disease; LIP, lymphoid interstitial pneumonia; NSIP, non-specific interstitial pneumonia; OP, organising pneumonia; PD, pulmonary disease; SLS, shrinking lung syndrome; UIP, usual interstitial pneumonia.

The prevalence of PD increased to 78%, when adding information concerning SLS, pleuritis according to SLICC definition,^28^ asthma, lung cysts, lung nodules from patients’ interview, review of medical records and HRCT to the diagnoses from MDD. According to patient’s interview and review of medical records, 4 (2.2%) had SLS and 99 (54%) had pleural disease. HRCT showed that 16 (8.7%) had cysts and 22 (12%) nodules (online supplemental Appendix 3).

### Cumulative incidence proportions

**Figure 2:**
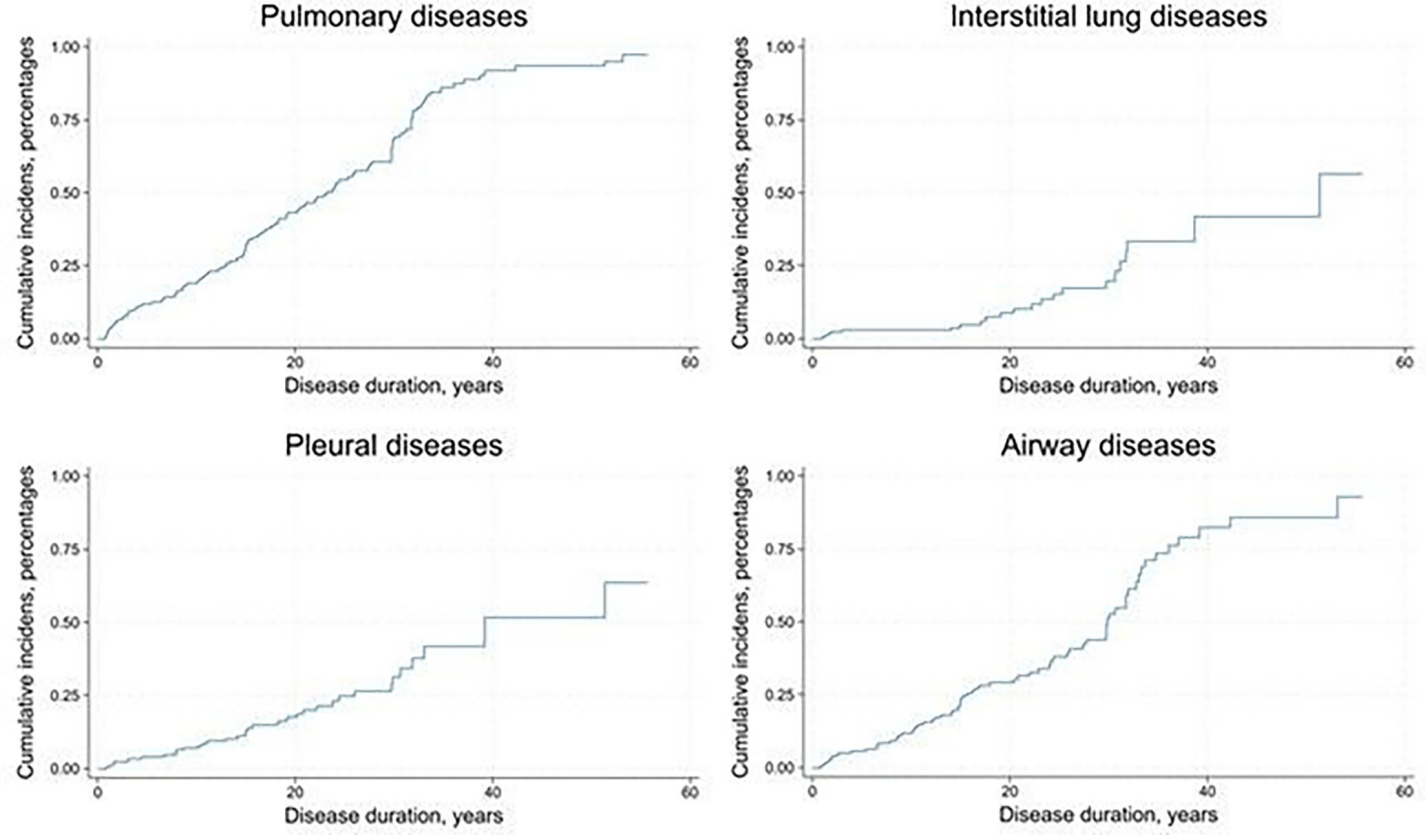
Cumulative incidence proportions of pulmonary diseases and subtypes of pulmonary diseases in 185 patients with SLE according to disease duration.

The cumulative incidence proportion for PD and its subtypes showed a linear progression over time. We observed an additional increase after approximately 30 years of disease duration, with the exception of ILD, which demonstrated an initial increase, followed by a plateau, then a rise after 17 years (figure 2).

### Associations to PD

In univariable regression analyses, PD was associated with age, self-reported breathlessness using MRC and several PFT measures (table 3 and online supplemental Appendix 4). The subtypes of PD were also associated with certain variables in univariable regression analyses: ILD with increasing age, overlap with other ARD, prior pleuritis, increasing SDI, a prior ‘chest tightness’ and several PFT measures; pleural diseases with overlap to other ARD, APS, increasing SDI and several PFT measures; airway diseases with increasing age, increasing SDI and several PFT measures (table 3 and online supplemental Appendix 4). Smoking was not associated with PD or its subtypes (table 3).

**Table 3:** Impact of selected baseline characteristics and patient related outcome measures in PDs among 185 patients with SLE investigated by univariable logistic regression analyses

Baseline characteristics	PD OR (95% CI)	ILD OR (95% CI)	Pleural diseases OR (95% CI)	Airway diseases OR (95% CI)
Age, years	1.05 (1.03 to 1.08)	1.06 (1.03 to 1.10)	1.01 (0.989 to 1.04)	1.03 (1.01 to 1.06)
Female sex, n=160/185 (87%)	0.95 (0.40 to 2.2)	0.67 (0.21 to 2.2)	0.92 (0.32 to 2.7)	1.1 (0.46 to 2.6)
Disease duration, years	0.97 (0.95 to 0.999)	0.97 (0.94 to 1.0)	1.0 (0.97 to 1.03)	0.98 (0.95 to 1.00)
Smoking, status, n=185	0.33*	0.86 *	0.54 *	0.20 *
Never, n=86 (46%)	0.72 (0.40 to 1.3)	1.2 (0.48 to 2.9)	1.3 (0.66 to 2.7)	0.60 (0.33 to 1.1)
Prior, n=80 (43%)	1.6 (0.86 to 2.9)	1.4 (0.56 to 3.3)	0.98 (0.47 to 2.1)	1.7 (0.94 to 3.1)
Present, n=19 (10%)	0.75 (0.29 to 2.0)	Empty	0.47 (0.10 to 2.2)	0.95 (0.36 to 2.6)
Pleuritis, positive†, n=99/185 (54%)	/	3.4 (1.2 to 9.5)	/	1.1 (0.57 to 1.9)
dsDNA positive ever, n=165/185 (89%)	1.2 (0.47 to 3.1)	1.2 (0.27 to 5.8)	0.93 (0.29 to 3.0)	1.5 (0.54 to 4.0)
SLEDAI 2K, score 0–105	0.92 (0.85 to 1.0)	0.85 (0.72 to 1.0)	1.0 (0.90 to 1.1)	0.98 (0.89 to 1.11)
SDI, score 0–48	1.50 (1.2 to 1.8)	1.4 (1.2 to 1.7)	1.4 (1.2 to 1.6)	1.2 (1.0 to 1.3)

* P values were calculated using the χ² test.

† Defined as pleural disease according to definition in SLICC 2012 classification criteria. Planned univariable analysis.

dsDNA, double stranded DNA antibodies; ILD, interstitial lung disease; PD, pulmonary disease; SDI, Systemic Lupus International Collaborating Clinics/American College of Rheumatology Damage Index for SLE; SLEDAI 2K, Systemic Lupus Erythematosus Disease Activity Index 2000; SLICC, Systemic Lupus International Collaborating Clinics.

In multivariable analyses (online supplemental Appendix 6a-d) PD were associated with increasing age, increasing SDI and decreased SDI no-PD, and in a fitted multivariable analysis PD were associated with increasing age, positive antiphospholipid antibodies and reduced DLCO; ILD were associated with increasing age, increasing SDI and decreased SDI no-PD and in a fitted multivariable analysis with increasing age, increasing SDI and reduced FVC; pleural diseases were associated with increasing SDI, decreased SDI no-PD, APS and reduced DLCO, and in a fitted multivariable analysis with increasing age and APS; airway diseases were associated with reduced TLC and in a fitted multivariable analysis to reduced TLC and reduced FEV_1_/FVC ratio.

### Pulmonary function tests

PD and its subtypes were significantly associated with abnormal PFT measures (online supplemental Appendix 2a-d and online supplemental Appendix 4). Reduced lung volumes (measured as low FVC and TLC) and DLCO were associated with ILD and pleural diseases, while lower FEV1/FVC ratios were more closely associated with airway diseases (online supplemental Appendix 2b-d). Participants with PD reported significantly more breathlessness according to MRC (online supplemental Appendix 1), and increased MRC was associated with ILD and airway diseases in univariable analyses (online supplemental Appendix 4). Results of the 6MWT were not associated with PD or its subtypes except pleural disease and decrease in oxygen saturation (online supplemental Appendix 2a-d and online supplemental Appendix 4). PFT showed balanced accuracies of 44%–63% across the different PD subtypes (online supplemental Appendix 5).

## Discussion

In this population-based, cross-sectional study, we show that PDs were prevalent in more than half of patients with SLE, and the most common PD subtypes were ILD, pleural diseases and airway diseases. PD and subtypes of PD seem important as they were associated with reduced PFT measures and breathlessness. In multivariable regression analyses, we found PD and its subtypes were associated with increasing age, increasing SDI and reductions in different PFT measures.

PD was present in 59% of participants, and the prevalence rose to 78% if additional medical history and minor changes on HRCT were included. This broader definition might overestimate the presence of PD in SLE but has been used in other studies addressing PD in SLE.^9 29 30^ According to expert opinion, the prevalence of PD in SLE is approximately 50%^2 3^ while studies with different designs report prevalences ranging from 30% in a register-based study,^5^ 56% in a dedicated clinical study,^31^ to 98% in an autopsy study.^7^ PD was associated with several decreased PFT measures and participants’ sensation of breathlessness, underscoring that PD is of clinical importance in SLE and suggesting MRC is a relevant screening tool. We found no association between PD and lower scores for HRQL, consistent with one study^31^ but contrary to another.^6^ Interestingly, smoking was not associated with PD; this was also true for all subtypes. This could be due to recall bias or to minor impact of smoking on PD in SLE.

ILD draws much attention in ARD in general, but in SLE, ILD may be overshadowed by more obvious manifestations, for example, from skin and kidneys. This is indicated by the lack of studies as a basis for screening, diagnosing and treating SLE-ILD, according to the latest ARD-ILD 2025 guideline by European Respiratory Society (ERS) and EULAR.^32^ One systematic review addressing the prevalence of SLE-ILD (defined as CT scan abnormalities) reported a prevalence of 6% based on three peer-reviewed studies^33^ and the ERS/EULAR 2025 guideline stated that SLE-ILD is rare.^32^ However, other studies of SLE-ILD^8 9 31^ report prevalences from 11% to 38%*.* We found 22 (12%) of the participants had ILD, including cases of progressive fibrosing ILD. The SLE-ILD cases seem clinically important, as they were associated with decreased PFT measures (online supplemental Appendix 2b) and breathlessness (online supplemental Appendix 1). SLE-ILD was more prevalent among participants with overlap to other ARD, but not to specific other ARD (online supplemental Appendix 4). Since SLE-ILD was associated with higher SDI, older age, sensation of breathlessness, abnormal PFT measures and pleuritic pain in univariable analyses, they are all potential risk markers of SLE-ILD, but only increasing SDI performed well in multivariable analysis (online supplemental Appendix 6b). Because no variables were strongly associated with SLE-ILD and PFT seemed insufficient to identify all patients at risk of SLE-ILD, HRCT is currently necessary to identify SLE-ILD.

Pleural diseases are thought to be the most common pulmonary manifestations in SLE, and approximately 50% of the patients with SLE report a history of pleural diseases.^2 3^ We found that pleural diseases were prevalent in 35 (19%) of the participants, increasing to 99 (54%) if we included patients with pleural serositis according to the SLICC classification criteria.^28^ Pleural diseases were associated with several variables, most strikingly APS, several reduced PFT measures predominantly with a restrictive pattern (online supplemental Appendix 6c) and increasing SDI. Note pleural diseases are included in SDI. We speculate whether APS, by involvement of small vessels, could cause pleural inflammation like in pulmonary embolism (PE), which is often associated with pleurisy, effusion and inflammation,^34^ or whether APS and pleural diseases share another non-vascular pathogenesis.

Lower airway diseases are estimated to affect approximately 20% of patients with SLE,^3^ based on few studies. In this study, airway diseases, including bronchiectasis, were the most common subtype of PD affecting 70 (38%) of the participants. Participants with airway diseases had higher age, higher SDI score and decreased PFT mostly in an obstructive pattern (online supplemental Appendix 6d). Airway diseases were associated with a sensation of breathlessness measured by MRC, why this instrument might help to increase awareness of airway diseases in SLE. Lower airway diseases are associated with infections,^35^ and considering that infections are one of the leading causes of death in SLE,^36^ and that the respiratory tract is the most common site of infections in SLE,^7 37^ the importance of diagnosis of airway diseases in SLE should be emphasised.

SLS is a rare but serious PD in SLE estimated to affect 1% of patients with SLE,^3^ but a larger prevalence has been reported.^38^ We found one participant with SLS, but uncertainty is indicated by the 95% CI (0.08% to 3.8%). In our review of the medical records, additionally, three participants were classified as having SLS, adding to a total of 4 (95% CI 0.8 to 5.7%). The number of cases is in accordance with prior estimates^3^ but lower than in the observational study.^38^ This might reflect our stringent criteria for diagnosing PD, including SLS, and why mild/uncertain cases of SLS might be undiagnosed.

The cumulated incidence analyses indicated PD may develop at any time during patients’ disease course. SLE-ILD was commonly diagnosed within 2 years of SLE diagnosis, a finding in accordance with a French epidemiological study,^39^ and the prevalence of SLE-ILD increased among participants with more than 17 years of SLE disease duration. Information about long disease duration is based on small numbers and the cumulative incidence analyses were performed post hoc.

Even though abnormal pulmonary function was associated with PD and its subtypes, PFT was not sufficient for identification of participants with PD (online supplemental Appendix 5). Contrary, an abnormal PFT should give rise to additional investigations for PD, whereas a normal PFT does not exclude PD. No measures from the 6MWT were associated with PD or its subtypes, except for a decrease in oxygen saturation and presence of pleural diseases, indicating that 6MWT should not be used to diagnose PD in SLE. It is noticeable that 46% of the participants without PD had decreased DLCO (online supplemental Appendix 2a), in line with prior studies.^40 41^ Decreased pulmonary diffusion can be caused by many conditions including ILD, pulmonary hypertension (PH) and PE.^42^ The present study was unfortunately not directed towards diagnosing PH and PE, but other studies report the prevalences of PH and PE to be less than 10% in patients with SLE,^3^ so other causes of low DLCO should be considered. We speculate if endothelial dysfunction contributes to decreased DLCO, as higher levels of cytokines associated with endothelial dysfunction are common in SLE^43^ and in other patients with a decreased DLCO.^44^ In a small SLE cohort, decreased DLCO did not predict future PD and improved during 9 years follow-up.^45^ Our results demonstrate that a decreased DLCO is common in SLE even without underlying PD, which is why DLCO should not be used to evaluate presence of PD. However, the cause of decreased DLCO in SLE remains unclear, to our knowledge.

Although some abnormalities on HRCT were not considered a disease, it is noteworthy that 22 (12%) had nodules and 16 (8%) had cysts on HRCT (online supplemental Appendix 3). Cysts have been reported in 16% of patients with SLE^46^ and nodules in 22% of asymptomatic patients with SLE,^30^ showing that these are common findings in SLE.

Our study has several strengths. In a population-based design, we included 185 well-characterised patients with SLE, meeting relevant classification criteria.^12^ 58% of the invited participated with only small differences between participants and non-participants regarding age and sex. Participants underwent a comprehensive diagnostic workup for various PD and were diagnosed according to gold standard.^26^ Before initiation, the study was registered at clinicaltrials.org, and we developed an SAP prior to the data analyses. However, the study was cross-sectional, which is why investigations of the development of PD are limited. Despite the participants undergoing a thorough diagnostic workup, the study was not designed to diagnose some specific diseases such as asthma, PH and PE, and prior PD might be undiagnosed. The study could be affected by ascertainment bias, as patients believing they had PD might have been more likely to participate. Our study population differed from others as participants were older, had longer disease duration, had lower disease activity and were predominately white.^47^ The composition of the study population probably had little effect on the prevalences, as white ethnicity and low disease activity are associated with reduced damage accrual,^48^ whereas older age is associated with increased damage and PD.^49^ However, the composition of the study population must be considered when generalising our results.

As PD is frequent and of clinical importance in SLE, the surveillance of SLE disease activity should include attention towards PD. Patient reporting, including MRC, may help identify patients at risk together with higher age and SDI. At present, HRCT is a key component for diagnosis of PD at MDD, but biomarkers and other imaging modalities, for example, thoracic ultrasound, could potentially contribute to the diagnostic process. Another important future scientific focus will be to investigate the underlying mechanisms of decreased DLCO unrelated to PD in SLE.

## Supplementary material

10.1136/lupus-2025-001895 online supplemental file 1

10.1136/lupus-2025-001895 online supplemental file 2

10.1136/lupus-2025-001895 online supplemental file 3

10.1136/lupus-2025-001895 online supplemental file 4

10.1136/lupus-2025-001895 online supplemental material 1

## Data Availability

Data are available upon reasonable request.
