# Supplementary material for: Pulmonary diseases in SLE: a population-based cross-sectional study
Source: Lupus Sci Med. 2026 Mar 31;13(1):e001895. doi: 10.1136/lupus-2025-001895 (PMC13052775; doi:10.1136/lupus-2025-001895)
Supplement: online supplemental file 1 [file lupus-13-1-s001.pdf]

Participants were scanned with a Revolution CT; GE; Boston, Massachusetts, USA. Acquisition parameters of the diagnostic end-inspiratory scanning are collimation 8 cm, kV 120, SmartmA (140–900 mA), Noise Index 25, Pitch 0.5, Rotation time 0.35 s, Asir-V 40%. Images were reconstructed using a 1024×1024 matrix and chest algorithm. Axial slice thickness was 0.625 mm. Image overlap 20%. Almost identical acquisition parameters were used for the low-dose end-expiratory scanning, with noise index was raised to 30. End-expiratory images were reconstructed using a standard algorithm. Examinations were assessed on Vue PACS; Philips, Amsterdam, The Netherlands.
