# Supplementary material for: Pulmonary diseases in SLE: a population-based cross-sectional study
Source: Lupus Sci Med. 2026 Mar 31;13(1):e001895. doi: 10.1136/lupus-2025-001895 (PMC13052775; doi:10.1136/lupus-2025-001895)
Supplement: online supplemental file 2 [file lupus-13-1-s002.pdf]

Statistical Analysis Plan (SAP)

Prevalence and Types of Concomitant Pulmonary Disease in Systemic Lupus Erythematosus: A Population-Based Cross-Sectional Study

1. Administrative information

Ethics committee number: S-20210164

Data protection agency number: 23/22079

Clinicaltrials.gov number: NCT06087523

SAP plan number: v.0.2025-0207

Date: 2025, February 24th

Roles

Statistical analyst and writer of statistical analysis plan. Henrik Zachar Langkilde, MD

Statistical advisor. Robin Christensen; BSc, MSc, PhD; Professor of Biostatistics & Clinical Epidemiology

Principle investigator. Anne Voss, MD, PhD; Assoc. Professor of Rheumatology

Collaborators

Jesper Rømhild Davidsen, MD, PhD, Professor of Respiratory Medicine

Stefan Harders, MD, PhD, Associate Professor of Radiology

Stefan Luef, MD

Susan Due Kay, MD, PhD

Elisabet Svenungsson, MD, PhD, Professor of Rheumatology

Sille Fløjborg, Patient Partner

Tine Lottenburger, MD, PhD

Stavros Chrysidis, MD, PhD, Associate Professor of Rheumatology

Karen Schreiber, MD, PhD, Associate Professor of Rheumatology

Signatures

Henrik Zachar Langkilde

Date and signature: 24/2-2025 [illegible]

Robin Christensen 2025-Feb-24

Date and signature: [illegible]

Anne Voss

Date and signature: 24/2 '25 [illegible]

Purpose of the Statistical Analysis Plan

In the following, we describe the statistical analysis plan (SAP) for a cross-sectional study on pulmonary disease among patients with systemic lupus erythematosus in the Southern Denmark Region. The purpose of this SAP is to outline our planned analyses for the study "Prevalence and Types of Concomitant Pulmonary Disease in Systemic Lupus Erythematosus: A Population-Based Cross-Sectional Study." Given the observational nature of the study and the large number of variables, we consider it essential to ensure transparency in our analytical approach. The study has been registered at ClinicalTrials.gov (NCT06087523), but no protocol has been published. Data on pulmonary disease (PD) diagnoses and high-resolution computed tomography (HRCT) evaluations are stored in a Research Electronic Data Capture (REDCap) database and remain blinded to the statistician, principal investigator, and statistical advisor until this statistical analysis plan is finalized and signed.

INTRODUCTION

Systemic Lupus Erythematosus (SLE) is a systemic autoimmune disease that may affect any organ. SLE is mostly associated with haematological abnormalities, rashes, arthritis/arthralgia, nephritis, and constitutional symptoms (1). Pulmonary diseases are common among SLE patients and affect up to 70% of the patients (2, 3), but the prevalence of PD among SLE patients are not investigated in a clinical study with a population-based design, to our knowledge. Prior studies have mostly investigated specific subgroups of PD and not PD in general. The PD commonly associated to SLE are airway disease, pleural disease, interstitial lung diseases (ILD), vasculitis, pulmonary haemorrhage, pulmonary hypertension, and the syndrome "Shrinking Lung Syndrome" (2-4).

Pulmonary Disease among SLE patients are important, as they are associated to an increased mortality (5) and decreased health-related quality of life (6). Unfortunately, there are a substantial difference between how patients report pulmonary symptoms and how physicians rate PD (7). It can be difficult to diagnose PD among SLE patients, for instance ILD is reported with a prevalence of 11-37% in clinical studies at tertiary centres (8-11) but only around 6% in register studies (12). To diagnose PD among SLE patients, healthcare providers need to have suspicion. We believe that a detail knowledge of how common different PD are among SLE patients will help guide physicians to be aware of which PD they should suspect and maybe subsequently investigate. Importantly, a study have shown, that immunosuppression can improve pulmonary function among SLE patients (13).

It is not always clear if pleura, pulmonary vasculature, and diaphragm are covered by the term "pulmonary disease". In the following we will refer to diseases of the pleura, airways, pulmonary vasculature, diaphragm, and lung parenchyma as pulmonary diseases (PD).

Objectives

The primary objective is to determine the prevalence of concomitant PD among SLE patients in a prospectively designed cross-sectional study.

Secondary objectives, based on the same primary sample of SLE patients to evaluate the:

- Baseline characteristics and outcome measures, overall and stratified by the presence or absence of PD
- Pulmonary function test (PFT) results, overall and stratified by the presence or absence of PD
- Prevalence of specific PD subtypes
- Associations between baseline characteristics and outcome measures and the presence of PD and selected pre-defined subtypes of PD

METHODS

The present study is part of a larger research initiative. Findings on the associations between PD and PD subtypes and pulmonary function test (PFT) measures, thoracic ultrasound, diaphragmatic ultrasound, and additional biomarkers will be reported in separate publications. A separate study will examine PD diagnoses and selected outcomes, including thoracic and diaphragmatic ultrasound, in newly diagnosed systemic lupus erythematosus (SLE) patients. Patients were identified through a systematic search of hospital records across all institutions in the Southern Denmark Region. The search includes visits between July 1, 2021, and June 30, 2023, coded as related to SLE or SLE-associated diseases. Participants were enrolled between August 1, 2023, and October 31, 2024. Statistical analyses will commence upon finalization and approval of this SAP.

At a single visit day all tests were performed. All participants start with an outpatient visit, where the primary investigator review the participants' medical history, note the patients' disease activity and damage. Hereafter the participants undergo pulmonary function testing, HRCT scan, and blood and urine samples. Questionnaires are filled out before the visit. The HRCT scans are described after the visits, and finally the participants are diagnosed, at a multidisciplinary discussion (MDD). The study will be reported in accordance with the STROBE guideline (Strengthening of Reporting Observational Studies in Epidemiology). It is not always possible to measure a precise saturation in a six-minute walk test, why we have scored them as good, medium, poor, too bad to use. Good is tests without any outages. Medium is tests with outages, that which we do not suspect affect the measurement. Poor is tests where we suspect, that the outages affect the results. We will only use tests that score good or medium, and the rests will be regarded as missing values.

Statistical analyses

Analysis populations

The primary analyses will be based on the Intention to Survey (ITS) population (14). This principle assesses the effect of all participants investigated for PD stratified by the presence of PD and subtypes of PD. Accordingly, participants allocated to a PD group will be assessed and analysed as members of that group, irrespective of the availability of the various collected characteristics and outcome variables.

Baseline patient characteristics: Descriptive statistics

Mock-up Table 1. Descriptive statistics at baseline in a study like the present summarize the key characteristics of the study population to provide an overview before any interventions or outcomes are analysed. These statistics typically include demographic characteristics (e.g., age, sex, ethnicity), clinical characteristics (e.g., comorbidities, medication use), lifestyle factors (e.g., smoking status), and laboratory data (e.g., blood samples). Continuous variables will be summarized by means and standard deviations (SD) or medians and interquartile ranges (IQR), while categorical variables will be presented as frequencies and percentages. When the cross-sectional sample is divided into subgroups (e.g., PD vs. No PD), baseline characteristics will be compared using standardized differences (Std. Difference) to assess balance across groups; they are independent of sample size, allowing for a consistent measure of covariate imbalance without being influenced by statistical significance. These descriptive statistics will be presented in tables for clarity, and their purpose is to characterize the cohort, assess comparability between groups, and provide context for interpreting the study's results. Interpretation of standardized differences include <0.1 indicates a negligible difference; values between 0.1 and 0.2 might suggest a small difference, and >0.2 may indicate an imbalance.

Comparing the SLE population: Analysis methods comparing PD exposed with PD-unexposed

Mock-up Table 2. We will compare baseline characteristics across SLE strata, including Systemic Lupus Erythematosus activity and damage scores, patient-reported outcome measures, and pulmonary function tests, for all patients and by the presence or absence of thoracic disease. All 95% confidence intervals (95%CI) will be two sided. The analyses of the secondary outcomes will be performed in sequence. The 95%CI will not be adjusted for multiplicity and should not be used in place of hypothesis testing.

Prevalence of groups and subgroups of pulmonary diseases: Descriptive statistics

Mock-up Table 3. We will describe the prevalence of specific groups (e.g. airway disease, ILD) and subgroups (e.g. asthma, bronchiectasis, atelectasis) of PD among the study participants. We plan to present the prevalence with absolute numbers and proportion as percentages.

Logistical regression: Searching for risk factors that are associated with presence of different forms of PD

Mock-up Table 4. We aim to explore whether one or more clinical measures collected at baseline are associated with the presence of PD. The dependent variable will be the presence or absence of a PD condition of interest (i.e., Y=1 if yes, otherwise Y=0). Logistic regression is the best approach for identifying predictors when the outcome variable is binary (i.e., presence vs. absence of PD) and the goal is to estimate the association between predictor variables and the probability of the outcome while enabling adjustments for potential confounders. Independent variables, as listed in Table 4 and appendix 3, will be analysed in univariate logistic regression to identify potential associations with clinically significant PD conditions. Odds ratios (ORs) with 95%CIs will be used to quantify the strength of associations, with 95%CIs excluding 1 considered indicative of potential significance. In multiple logistic regression analyses we plan to investigate associations that were significant by univariate analyses, however, only a subset of independent variables will be expected to remain statistically significant and independently associated with PD conditions.

Missing data

We expect missing data to be Missing Completely At Random. We plan to investigate the possible impact of missing data in sensitivity analyses. Multiple Imputation (MI) is a statistical technique used to handle missing data by creating multiple plausible datasets, analysing each separately, and then pooling the results to account for uncertainty. This approach is particularly useful for sensitivity analyses in cross-sectional descriptive studies, ensuring that findings are robust to different assumptions about missing data. We will also explore the impact of the missing data by applying a best- and worst-case scenario.

Statistical software

We will analyse the results with StataBE 18.0 (64bit).

RESULTS

In the following we show, how we plan to report the results.

We plan to present the trial flow with a flowchart as described in figure 1.

Figure1. Flowchart describing the trial flow of possible participants in the study.

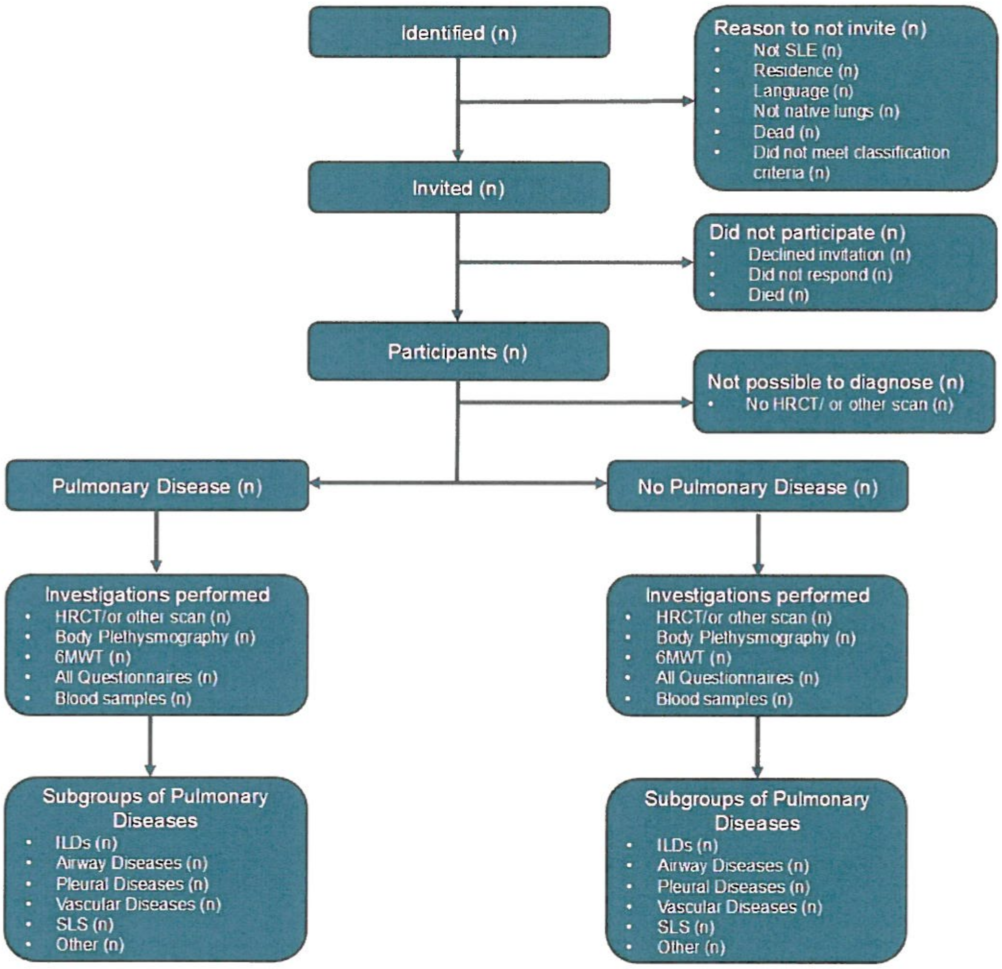

SLE – Systemic Lupus Erythematosus; HRCT – High Resolution CT scan; 6MWT – Six Minute Walk Test; ILDs – Interstitial Lung Diseases; SLS – Shrinking Lung Syndrome.

We plan to present baseline characteristics, Systemic Lupus Erythematosus activity and damage scores, patient reported outcome measures, and pulmonary function tests, for all patients and stratified according to presence and absence of PD and analyses of differences in the variables according to status of PD, in two tables, see table 1 and table 2.

Table 1. Baseline characteristics, for all patients, and stratified according to presence and absence of thoracic diseases

Baseline characteristics	All	PD Present	PD Absent	Std. Difference
Age, years				
Disease duration, years				
Female sex, no. (%)				
White population no. (%)				
Smoking, composite				
Smoking, status<br>Never, no. (%)<br>Prior, no. (%)<br>Present, no. (%)				
BMI, kg/m^2				
Overlap to other CTDs, composite				
Overlap to other CTDs, no. (%)<br>Sjogren's Syndrome, no. (%)<br>Rheumatoid Arthritis, no. (%)<br>Systemic Sclerosis, no. (%)<br>Mixed Connective Tissue Disease, no. (%)<br>Antiphospholipid Syndrome, no. (%)				
Treatment:				
Corticosteroid ever, no. (%)				
Corticosteroid current, no. (%)				
Hydroxychloroquine ever, no. (%)				
Hydroxychloroquine current, no. (%)				
Other csDMARDs ever, no. (%)				
Other csDMARDs current, no. (%)				
BioDMARDs ever, no. (%)				
BioDMARDs current, no. (%)				
Clinical findings:				
EULAR/ACR 2019 classification criteria, score 0-51				
Constitutional*, score 0-2				
Haematological*, score 0-4				
Neuropsychiatric*, score 0-5				
Mucocutaneus*, score 0-6				
Serosal*, score 0-6				
Musculoskeletal*, score 0-6				
Renal*, score 0-4				
Blood samples:				
APL positive* ever, score 0-2				
Complement low* ever, score 0-4				
dsDNA /Smith elevated* ever, score 0-6				
SLICC 2012 classification criteria, score 0-17				
ACR 1997 classification criteria, score 0-11				

Value will be means and SDs unless otherwise indicated. *According to EULAR/ACR 2019 classification criteria (15). PD – Pulmonary Disease according to multidisciplinary discussion; BMI – Body Mass Index; CTD – Connective tissue disease; csDMARD – Conventional Disease-Modifying Antirheumatic Drugs; bioDMARDs – Biologic Disease-Modifying Antirheumatic Drugs. APL– Antiphospholipid Antibodies; dsDNA – anti-double stranded DNA antibodies. The reported Standardised

Difference is the difference between PD present and PD absent, standardised deviation based on the standard deviation for continuous outcomes measures.

Table 2. Systemic Lupus Erythematosus activity and damage scores, patient reported outcome measures, pulmonary function tests, for all patients and stratified according to presence and absence of pulmonary disease.

SLE scores, PROMs, and PFT outcomes	All	PD Present	PD Absent	Contrast between groups (95%CI)
SLE disease specific score:				
SLEDAI 2K, score 0-105				
SLEDAI 2K, without PD, score 0-103				
PGA, score 0-3				
LLDAS achieved no. (%)				
DORIS remission achieved no. (%)				
SLICC DI, score 0-48				
SLICC DI, without PD, score 0-43				
PROMs:				
LIT, score 0-100				
SLAQ, score 0-100				
MRC, score 1-5				
Dry cough, composite				
Dry cough, status<br>Never, no. (%)<br>Prior, no. (%)<br>Present, no. (%)				
Tightness in chest, composite				
Tightness in chest, status<br>Never, no. (%)<br>Prior, no. (%)<br>Present, no. (%)				
**Pulmonary function tests:**				
Body plethysmography:				
FEV_1, %*				
FEV_1 low**, no. (%)				
FVC, %*				
FVC low**, no. (%)				
FEV_1/FVC (ratio)				
FEV_1/FVC below 70%, no. (%)				
TLC, %*				
TLC low**, no. (%)				
Diffusion test:				
DLCO, %*				
DLCO low**, no. (%)				
KCO, %*				
KCO low**, no. (%)				
6 min walk test:				
Distance, %*				
Distance low**, no. %				
Desaturation, (Saturation delta)				
Desaturation below 93% #, no. %				

Value will be means and SDs unless otherwise indicated. * - % of expected; ** - low is defined as below 80% of expected; # - If COPD or emphysema 88%. SLE – Systemic Lupus Erythematosus; PROMs – Patient Reported Outcome Measures; PFT – Pulmonary Function Tests; PD – Pulmonary Disease according to multidisciplinary discussion; APL– Antiphospholipid Antibodies; dsDNA – anti-double stranded DNA antibodies; SLEDAI 2K – Systemic Lupus Erythematosus Disease Activity Index 2000 (16); PGA – Physician Global Assessment (17); LLDAS – Lupus Low Disease Activity State (18); DORIS – Definition of

Remission in SLE (19); SLICC DI – Systemic Lupus International Collaborating Clinics /American College of Rheumatology Damage Index for Systemic Lupus Erythematosus (20); LIT – questionnaire inspired by "Lupus Impact Tracker" (21); SLAQ – Systemic Lupus Activity Questionnaire (22); MRC – Medical Research Council Dyspnoea Scale; FEV1 – Forced Expiratory Volume in One Second; FVC – Forced Vital Capacity; TLC – Total Lung Capacity; DLCO - Diffusing capacity of the Lung for Carbon Monoxide; KCO – Carbon Monoxide Transfer Coefficient.

We plan to present the prevalence of subgroups of PD in a table, see table 3.

Table 3. Diagnoses of pulmonary diseases among Systemic Lupus Erythematosus patients.

Groups of PD	Subgroups of PD	Prevalence, no. (%)
Airway		
	Asthma	
	Bronchiectasis	
	Obliterative Bronchiolitis	
	Infectious Bronchiolitis	
	COPD	
	Emphysema	
	Other	
Pleural		
	Pleuritis	
	Pleural disease of diaphragm	
	Pleuroparenchymal fibrosis	
	Other	
ILD		
	UIP	
	Probable UIP	
	Indeterminate for UIP	
	NSIP, fibrotic	
	NSIP, non-fibrotic	
	HP fibrotic	
	HP non-fibrotic	
	Pneumonitis	
	Diffuse alveolar haemorrhage	
	LIP	
	OP	
	Other	
SLS		
Vascular		
	Pulmonary embolism	
	Pulmonary arterial hypertension	
	Pulmonary vasculitis	
	Other	
Other		
	Atelectasis	
	Infections	
	Thoracic malignancy	
	Other	

PD – Pulmonary Disease; COPD – Chronic Obstructive Pulmonary Disease; UIP – Usual Interstitial Pneumonia; NSIP – Non-Specific Interstitial Pneumonia; HP – Hypersensitivity Pneumonitis; LIP – Lymphoid Interstitial Pneumonia; OP – Organising Pneumonia; SLS – Shrinking Lung Syndrome. The percentage is of the total study population.

We plan to present results of PFT test of subtypes of PD (Airway disease, Pleural Disease, ILD, and SLS) and the contrast between groups with means, SD, and a 95% confidence interval for continues data or number and percentages for categorical data in appendixes, see Appendix 1 a-d.

We plan to present the prevalence of High-Resolution CT findings among the investigated study population is presented in an appendix, see appendix 2.

We plan to present results of the univariate logistic analyses, with Odds Ratio and corresponding 95%CI, of the association between selected baseline characteristics and the presence of PD, airway disease, pleural disease, ILD, and SLS in a table, see table 4.

Table 4. Impact of selected baseline characteristics and patient related outcome measures in groups of thoracic diseases investigated by univariate logistic regression analyses.

**PD Outcome vs. Baseline characteristics**	**PD Odds Ratio (95%CI)**	**Airway Odds Ratio (95%CI)**	**Pleural Odds Ratio (95%CI)**	**ILD Odds Ratio (95%CI)**	**SLS Odds Ratio (95%CI)**
Age, years					
Female sex, no (%)					
Disease duration, years					
Smoking, composite					
Smoking, status<br>Never, no. (%)<br>Prior, no. (%)<br>Present no. (%)					
Overlap to other CTDs, composite, no. (%)					
Overlap to other CTDs, no. (%)<br>Sjogren's Syndrome, no. (%)<br>Rheumatoid Arthritis, no. (%)<br>Systemic Sclerosis, no. (%)<br>Mixed Connective Tissue Disease, no. (%)<br>Antiphospholipid Syndrome, no. (%)					
Pleuritis, positive, no. (%)	/		/		
Pleural effusion, no. (%)	/		/		
Positive APL ever, no. (%)					
Decreased complement ever, no. (%)					
dsDNA positive ever, no. (%)					
SLEDAI 2K, score 0-105					
SLEDAI 2K, without PD, score 0-103					
SLICC DI, score 0- 48					
SLICC DI, without PD, score 0-43					
Dry cough, composite					
Dry cough, status:<br>Never, no. (%)<br>Prior, no. (%)<br>Present, no. (%)					
Tightness in chest, composite, no. (%)					
Tightness in chest, status:<br>Never, no. (%)<br>Prior, no. (%)<br>Present, no. (%)					

Planned univariate analysis. PD – Pulmonary Disease according to multidisciplinary discussion; 95% CI – 95% Confidence Interval; Airway – Airway Disease according to multidisciplinary discussion; Pleural – Pleural Disease according to multidisciplinary discussion; ILD – Interstitial Lung Disease positive according to multidisciplinary discussion; SLS – Shrinking

Lung Syndrome according to multidisciplinary discussion; CTD – Connective Tissue Disease; APL– Antiphospholipid Antibodies; dsDNA – anti-double stranded DNA antibodies; SLEDAI 2K – Systemic Lupus Erythematosus Disease Activity Index 2000; SLICC DI – Systemic Lupus International Collaborating Clinics /American College of Rheumatology Damage Index for Systemic Lupus Erythematosus. The reported Odds Ratio and 95% confidence interval is between participants positive and negative in regards of PD / Airway / Pleural / ILD / SLS.

References

1. Kaul A, Gordon C, Crow MK, Touma Z, Urowitz MB, van Vollenhoven R, et al. Systemic lupus erythematosus. Nat Rev Dis Primers. 2016;2:16039.
2. Hannah JR, D'Cruz DP. Pulmonary Complications of Systemic Lupus Erythematosus. Semin Respir Crit Care Med. 2019;40(2):227-34.
3. Bendstrup E, Lynn E, Troldborg A. Systemic Lupus Erythematosus-related Lung Disease. Semin Respir Crit Care Med. 2024.
4. Kamen DL, Strange C. Pulmonary manifestations of systemic lupus erythematosus. Clin Chest Med. 2010;31(3):479-88.
5. Narváez J, Borrell H, Sánchez-Alonso F, Rúa-Figueroa I, López-Longo FJ, Galindo-Izquierdo M, et al. Primary respiratory disease in patients with systemic lupus erythematosus: data from the Spanish rheumatology society lupus registry (RELESSER) cohort. Arthritis Res Ther. 2018;20(1):280.
6. Osman HM, Abdel-Nasser AM, Kasem AH, Elameen NF, Omar GM. Pulmonary involvement: A potential independent factor for quality of life in systemic lupus erythematosus. Lupus. 2023;32(2):198-206.
7. Svenungsson E, Gunnarsson I, Illescas-Bäckelin V, Trysberg E, Jönsen A, Leonard D, et al. Quick Systemic Lupus Activity Questionnaire (Q-SLAQ): a simplified version of SLAQ for patient-reported disease activity. Lupus Sci Med. 2021;8(1).
8. Fidler L, Keen KJ, Touma Z, Mittoo S. Impact of pulmonary disease on patient-reported outcomes and patient-performed functional testing in systemic lupus erythematosus. Lupus. 2016;25(9):1004-11.
9. Şenkal N, Kıyan E, Demir AA, Yalçınkaya Y, Gül A, İnanç M, et al. Interstitial lung disease in patients with systemic lupus erythematosus: a cohort study. Turk J Med Sci. 2022;52(1):76-82.
10. Su F, Xiao W, Yang P, Chen Q, Sun X, Li T. Anti-neutrophil cytoplasmic antibodies in new-onset systemic lupus erythematosus. An Bras Dermatol. 2017;92(4):466-9.
11. Fenlon HM, Doran M, Sant SM, Breatnach E. High-resolution chest CT in systemic lupus erythematosus. AJR Am J Roentgenol. 1996;166(2):301-7.
12. Joy GM, Arbiv OA, Wong CK, Lok SD, Adderley NA, Dobosz KM, et al. Prevalence, imaging patterns and risk factors of interstitial lung disease in connective tissue disease: a systematic review and meta-analysis. Eur Respir Rev. 2023;32(167).
13. Traynor AE, Corbridge TC, Eagan AE, Barr WG, Liu Q, Oyama Y, et al. Prevalence and reversibility of pulmonary dysfunction in refractory systemic lupus: improvement correlates with disease remission following hematopoietic stem cell transplantation. Chest. 2005;127(5):1680-9.
14. Christensen R, Ranstam J, Overgaard S, Wagner P. Guidelines for a structured manuscript: Statistical methods and reporting in biomedical research journals. Acta Orthop. 2023;94:243-9.
15. Aringer M, Costenbader K, Daikh D, Brinks R, Mosca M, Ramsey-Goldman R, et al. 2019 European League Against Rheumatism/American College of Rheumatology classification criteria for systemic lupus erythematosus. Ann Rheum Dis. 2019;78(9):1151-9.
16. Gladman DD, Ibañez D, Urowitz MB. Systemic lupus erythematosus disease activity index 2000. J Rheumatol. 2002;29(2):288-91.
17. Buyon JP, Petri MA, Kim MY, Kalunian KC, Grossman J, Hahn BH, et al. The effect of combined estrogen and progesterone hormone replacement therapy on disease activity in systemic lupus erythematosus: a randomized trial. Ann Intern Med. 2005;142(12 Pt 1):953-62.
18. Franklyn K, Lau CS, Navarra SV, Louthrenoo W, Lateef A, Hamijoyo L, et al. Definition and initial validation of a Lupus Low Disease Activity State (LLDAS). Ann Rheum Dis. 2016;75(9):1615-21.
19. van Vollenhoven RF, Bertsias G, Doria A, Isenberg D, Morand E, Petri MA, et al. 2021 DORIS definition of remission in SLE: final recommendations from an international task force. Lupus Sci Med. 2021;8(1).

20. Gladman D, Ginzler E, Goldsmith C, Fortin P, Liang M, Urowitz M, et al. The development and initial validation of the Systemic Lupus International Collaborating Clinics/American College of Rheumatology damage index for systemic lupus erythematosus. Arthritis Rheum. 1996;39(3):363-9.

21. Jolly M, Garris CP, Mikolaitis RA, Jhingran PM, Dennis G, Wallace DJ, et al. Development and validation of the Lupus Impact Tracker: a patient-completed tool for clinical practice to assess and monitor the impact of systemic lupus erythematosus. Arthritis Care Res (Hoboken). 2014;66(10):1542-50.

22. Karlson EW, Daltroy LH, Rivest C, Ramsey-Goldman R, Wright EA, Partridge AJ, et al. Validation of a Systemic Lupus Activity Questionnaire (SLAQ) for population studies. Lupus. 2003;12(4):280-6.

Appendix 1a. Pulmonary function tests, according to prevalence of airway disease.

**Pulmonary function test measures.**	**N**	**Airway disease present**	**N**	**Airway disease absent**	**Contrast between groups (95%CI)**
Body plethysmography:					
FEV_1, %*					
FEV_1 low**, no. (%)					
FVC, %*					
FVC low**, no. (%)					
FEV_1/FVC (ratio)					
FEV_1/FVC below 70%, no. (%)					
TLC, %*					
TLC low**, no. (%)					
Diffusion test:					
DLCO, %*					
DLCO low**, no. (%)					
KCO, %*					
KCO low**, no. (%)					
6 min walk test:					
Distance, %*					
Distance low**, no. %					
Desaturation, (Saturation delta)					
Desaturation below 93% #, no. %					

Value will be means and SDs unless otherwise indicated. *- % of expected; ** - low is defined as below 80% of expected; # - If COPD or emphysema 88%; Airway disease positive – Airway Disease according to multidisciplinary discussion; FEV1 – Forced Expiratory Volume in One Second; FVC – Forced Vital Capacity; TLC – Total Lung Capacity; DLCO - Diffusing capacity of the Lung for Carbon Monoxide; KCO – Carbon Monoxide Transfer Coefficient. Contrasts for continuous measures will be based on the difference between means with 95% CIs, while dichotomous outcomes will be based on risk differences with 95% CIs.

Appendix 1b. Pulmonary function tests according to the prevalence of pleural disease.

**Pulmonary function test measures.**	**N**	**Pleural disease present**	**N**	**Pleural disease absent**	**Contrast between groups (95%CI)**
Body plethysmography:					
FEV_1, %*					
FEV_1 low**, no. (%)					
FVC, %*					
FVC low**, no. (%)					
FEV_1/FVC (ratio)					
FEV_1/FVC below 70%, no. (%)					
TLC, %*					
TLC low**, no. (%)					
Diffusion test:					
DLCO, %*					
DLCO low**, no. (%)					
KCO, %*					
KCO low**, no. (%)					
6 min walk test:					
Distance, %*					
Distance low**, no. %					
Desaturation, (Saturation delta)					
Desaturation below 93% #, no. %					

Value will be means and SDs unless otherwise indicated. * - % of expected; ** - low is defined as below 80% of expected; # - If COPD or emphysema 88%; Pleural disease present – Pleural Disease according to multidisciplinary discussion; FEV1 – Forced Expiratory Volume in One Second; FVC – Forced Vital Capacity; TLC – Total Lung Capacity; DLCO - Diffusing capacity of the Lung for Carbon Monoxide; KCO – Carbon Monoxide Transfer Coefficient. Contrasts for continuous measures will be based on the difference between means with 95% CIs, while dichotomous outcomes will be based on risk differences with 95% CIs.

Appendix 1c. Pulmonary function tests according to prevalence of Interstitial Lung Disease.

Pulmonary function test measures.	N	ILD present	N	ILD absent	Contrast between groups (95%CI)
Body plethysmography:					
FEV_1, %*					
FEV_1 low**, no. (%)					
FVC, %*					
FVC low**, no. (%)					
FEV_1/FVC (ratio)					
FEV_1/FVC below 70%, no. (%)					
TLC, %*					
TLC low**, no. (%)					
Diffusion test:					
DLCO, %*					
DLCO low**, no. (%)					
KCO, %*					
KCO low**, no. (%)					
6 min walk test:					
Distance, %*					
Distance low**, no. %					
Desaturation, (Saturation delta)					
Desaturation below 93% #, no. %					

Value will be means and SDs unless otherwise indicated. * - % of expected; ** - low is defined as below 80% of expected; # - If COPD or emphysema 88%; ILD – Interstitial Lung Disease; ILD present – ILD according to multidisciplinary discussion; FEV1 – Forced Expiratory Volume in One Second; FVC – Forced Vital Capacity; TLC – Total Lung Capacity; DLCO - Diffusing capacity of the Lung for Carbon Monoxide; KCO – Carbon Monoxide Transfer Coefficient. Contrasts for continuous measures will be based on the difference between means with 95% CIs, while dichotomous outcomes will be based on risk differences with 95% CIs.

Appendix 1d. Pulmonary function tests according to prevalence of Shrinking Lung Syndrome.

**Pulmonary function test measures.**	**N**	**SLS present**	**N**	**SLS absent**	**Contrast between groups (95%CI)**
Body plethysmography:					
FEV_1, %*					
FEV_1 low**, no. (%)					
FVC, %*					
FVC low**, no. (%)					
FEV_1/FVC (ratio)					
FEV_1/FVC below 70%, no. (%)					
TLC, %*					
TLC low**, no. (%)					
Diffusion test:					
DLCO, %*					
DLCO low**, no. (%)					
KCO, %*					
KCO low**, no. (%)					
6 min walk test:					
Distance, %*					
Distance low**, no. %					
Desaturation, (Saturation delta)					
Desaturation below 93% #, no. %					

Value will be means and SDs unless otherwise indicated. * - % of expected; ** - low is defined as below 80% of expected; ; # - If COPD or emphysema 88%; SLS - Shrinking Lung Syndrome; SLS present – Shrinking Lung Syndrome according to multidisciplinary discussion; FEV1 – Forced Expiratory Volume in One Second; FVC – Forced Vital Capacity; TLC – Total Lung Capacity; DLCO - Diffusing capacity of the Lung for Carbon Monoxide; KCO – Carbon Monoxide Transfer Coefficient. Contrasts for continuous measures will be based on the difference between means with 95% CIs, while dichotomous outcomes will be based on risk differences with 95% CIs.

Appendix 2. The prevalence of High-Resolution CT findings among Systemic Lupus Erythematosus patients.

Groups of HRCT findings	Subgroups of HRCT findings	N	Positive, no. (%)
Airways			
	Bronchiectasis		
	Bronchial wall thickening		
	Bronchiolitis		
	Air trapping		
	Other		
Pleural			
	Pleural thickening		
	Pleural effusion		
	Pleuroparenchymal fibrosis		
	Other		
Interstitial lung disease			
	UIP		
	Indeterminate for UIP		
	NSIP fibrotic		
	NSIP non-fibrotic		
	HP fibrotic		
	HP non-fibrotic		
	LIP		
	OP fibrotic		
	OP non-fibrotic		
	Other		
Elevated diaphragm			
Suspect of malignancy			
Emphysema			
Nodules			
Cysts			
Enlarged pulmonary arteries			
Other			

HRCT – High-Resolution CT scan; UIP – Usual Interstitial Pneumonia; NSIP – Non-Specific Interstitial Pneumonia; HP – Hypersensitivity Pneumonitis; LIP – Lymphoid Interstitial Pneumonia; OP – Organising Pneumonia. Percentage is of the total study population.

Appendix 3. Impact of baseline characteristics and patient related outcome measures that are not presented in table 4, according to groups of thoracic diseases investigated by univariate logistic regression analyses.

**Outcome measure / Baseline characteristic**	**PD OR (95%CI)**	**Airway OR (95%CI)**	**Pleural OR (95%CI)**	**ILD OR (95%CI)**	**SLS OR (95%CI)**
White population no. (%)					
BMI, kg/m^2					
Treatment:					
Corticosteroid ever, no. (%)					
Corticosteroid current, no. (%)					
Hydroxychloroquine ever, no. (%)					
Hydroxychloroquine current, no. (%)					
Other csDMARDs ever, no. (%)					
Other csDMARDs current, no. (%)					
BioDMARDs ever, no. (%)					
BioDMARDs current, no. (%)					
Classification criteria EULAR/ACR criteria, score 0-51,					
Constitutional*, score 0-2					
Haematological*, score 0-4					
Neuropsychiatric*, score 0-5					
Mucocutaneus*, score 0-6					
Serosal*, score 0-6					
Musculoskeletal*, score 0-6					
Renal*, score 0-4					
SLE disease specific score:					
SLEDAI 2K, score 0-105					
PGA, score 0-3					
LLDAS achieved no. (%)					
DORIS remission achieved no. (%)					
SLICC DI, score 0-48					
PROMs:					
LIT, score 0-100					
SLAQ, score 0-100					
MRC, score 1-5					
Body plethysmography:					
FEV_1, %^					
FEV_1 low˜, no. (%)					
FVC, %^					
FVC low˜, no. (%)					
FEV_1/FVC (ratio)					
FEV_1/FVC below 70%, no. (%)					
TLC, %^					
TLC low˜, no. (%)					
Diffusion test:					
DLCO, %^					
DLCO low˜, no. (%)					
KCO, %^					

KCO low˜, no. (%)					
6 min walk test:					
Distance, %^					
Distance low˜, no. %					
Desaturation (Saturation delta)					
Desaturation below 93% #, no. (%)					

Table of planned univariate analysis. *According to EULAR/ACR 2019 criteria (15). ˜ - low is defined as below 80% of expected; ^ - % of expected; # - If COPD or emphysema 88%. PD – Pulmonary according to multidisciplinary discussion; OR - Odds ration; 95% CI – 95% Confidence Interval; Airway – Airway Disease according to multidisciplinary discussion; Pleural – Pleural Disease according to multidisciplinary discussion; ILD – Interstitial Lung Disease positive according to multidisciplinary discussion; SLS – Shrinking Lung Syndrome according to multidisciplinary discussion; BMI – Body Mass Index; csDMARD – Conventional Disease-Modifying Antirheumatic Drugs; bioDMARDs – Biologic Disease-Modifying Antirheumatic Drugs; SLEDAI 2K – Systemic Lupus Erythematosus Disease Activity Index 2000 (16); PGA – Physician Global Assessment (17); LLDAS – Lupus Low Disease Activity State (18); DORIS – Definition of Remission in SLE (19); SLICC DI – Systemic Lupus International Collaborating Clinics /American College of Rheumatology Damage Index for Systemic Lupus Erythematosus; PROMs – Patient Reported Outcome Measures; LIT – questionnaire inspired by "Lupus Impact Tracker" (21); SLAQ – Systemic Lupus Activity Questionnaire (22); MRC – Medical Research Council Dyspnoea Scale; FEV1 – Forced Expiratory Volume in One Second; FVC – Forced Vital Capacity; TLC – Total Lung Capacity; DLCO - Diffusing capacity of the Lung for Carbon Monoxide; KCO – Carbon Monoxide Transfer Coefficient.

Deviations from SAP

In general:

Removed SLS from analysis, because only one was diagnosed with SLS why analyses were of limited value.

Some values have been changed in tables, to make them easier to understand.

Secondary objectives. Describe baseline characteristics has been removed, as it we did not find it was a relevant objective, but it is reported. Further, we have tried make the wording more concise to make them more readable.

Post hoc we developed Figure 2, Appendix 5, and fitted analyses. We included them, as they provided with important information.

Table 1 and Appendix 3 – in SAP

Removed subgroups of EULAR/ACR 2019, because it was more readable without the subgroups.

Table 1 – in SAP

The order of variables has been changed to make it more intuitive.

Removed ACR 1997, to make the table more readable, as we find the score outdated.

We included some important elements from Table 2.

Table 2 – in SAP

We removed Table 2, from the main text to avoid too much data in the main text and thereby make the manuscript more readable.

Some elements were moved to Table 1, the rest is moved to Appendix 1 or Appendix 2a.

Table 3 – in SAP

The table was moved up to become Table 2, because the former Table 2 was removed.

Pleuroparenchymal fibrosis changed to – sequelae of pleuritis. Because it was more consistent with current literature.

Others that were not present, was removed, to make the table more readable.

Table 4 -in SAP

The table was moved up to become Table 3, because the former Table 2 was removed.

Due to too much data of results some variables have been moved to Appendix 4 to make the table more readable.

Appendix 2 – in SAP now Appendix 3

ILD two new categories, ILA and Probable UIP. It was more informative to include the two groups.

Appendix 3 – in SAP now Appendix 4.

SLICC DI score is removed, as it is present I Table 3.
