# Supplementary material for: Pulmonary diseases in SLE: a population-based cross-sectional study
Source: Lupus Sci Med. 2026 Mar 31;13(1):e001895. doi: 10.1136/lupus-2025-001895 (PMC13052775; doi:10.1136/lupus-2025-001895)
Supplement: online supplemental file 3 [file lupus-13-1-s003.pdf]

Supplementary material C, clarification of others in table 3.

Other* diagnoses in table 3.

Interstitial Lung Diseases Other:

1 Interstitial Lung Abnormalities

1 Non-Specific Interstitial Pneumonia obs pro

1 CPFE – Combined Pulmonary Fibrosis and Emphysema

1 Sarcoidosis

2 Cysts

Pleural Diseases

2 Pleural thickening

1 Pleural adherences

Airway Diseases

4 Air trapping

1 Mucus plugging

Vascular Diseases

1 Aorta ectasia

Other

7 Nodules

2 Infiltrates

2 Resection

2 Infiltrates

1 Mediastinal mass

1 High diaphragm dexter

1 Cysts

1 Air trapping
