# Supplementary material for: Pulmonary diseases in SLE: a population-based cross-sectional study
Source: Lupus Sci Med. 2026 Mar 31;13(1):e001895. doi: 10.1136/lupus-2025-001895 (PMC13052775; doi:10.1136/lupus-2025-001895)
Supplement: online supplemental file 4 [file lupus-13-1-s004.pdf]

Appendix 1. SLE disease activity and damage scores, patient reported outcome measures, pulmonary function tests, for 185 patients with SLE, stratified according to presence or absence of pulmonary disease.

**Outcome Variables**	**All**	**PD Present**	**PD Absent**	**Contrast between groups (95%CI)**
SLE disease specific score:				
SLEDAI 2K, without PD, score 0-103 ^	4 (2-6)	2 (0-5)	4 (2-6)	2 (0.96 to 3.0)
PGA, score 0-3 ^	0.7 (0.4 to 1.0)	0.6 (0.4 to 1.0)	0.7 (0.4 to 1.0)	0.1 (-0.005 to 0.25)
LLDAS achieved no. (%)	124 (67%)	75 (69%)	49 (64%)	4.3% (-20% to 28%)
DORIS remission achieved no. (%)	69 (37%)	41 (38%)	28 (37%)	0.77% (-17% to 19%)
SDI, without PD, score 0-43 ^	1 (0-3)	1 (0-3)	1 (0-2)	0 (-0.69 to 0.69)
PROMs:				
LIT, score 0-100, (n= 181)	34.2 ± 18.4	33.5 ± 18.3	35.2 ± 18.8	1.74 (-3.8 to 7.3)
MRC, score 1-5 ^, (n = 182)	1 (1-2)	2 (1-2)	1 (1-2)	1 (0.57 to 1.4)
"Dry cough", status (n = 182)		n = 109	n = 73	0.36"
Never, no. (%)	123(68%)	71 (65%)	52 (71%)	6.1% (-18% to 31%)
Prior, no. (%)	12 (7%)	6 (5.5%)	6 (8.2%)	2.7% (-5.2% to 11%)
Present, no. (%)	47 (26%)	32 (29%)	15 (21%)	8.8% (-5.7% to 23%)
"Tightness in chest", status (n = 182)		n = 109	n = 73	0.32"
Never, no. (%)	100(55%)	58 (53%)	42 (58%)	4.3% (-18% to 26%)
Prior, no. (%)	17 (9.3%)	13 (12%)	4 (5.5%)	6.4% (-2.0% to 15%)
Present, no. (%)	65 (36%)	38 (35%)	27 (37%)	2.1% (-16% to 20%)

Without PD means the score without the components attributed to pulmonary diseases. Values are expressed as means and SDs unless otherwise indicated. ^ - Medians with interquartile range; " – p-value from chi-square test; Systemic Lupus Erythematosus; PROMs – Patient Reported Outcome Measures; PD – Pulmonary Disease according to multidisciplinary discussion; APL– Antiphospholipid Antibodies; dsDNA – anti-double stranded DNA antibodies; SLEDAI 2K – Systemic Lupus Erythematosus Disease Activity Index 2000 [16]; PGA – Physician Global Assessment [17]; LLDAS – Lupus Low Disease Activity State [18]; DORIS – Definition of Remission in SLE [19]; SDI – Systemic Lupus International Collaborating Clinics

/American College of Rheumatology Damage Index for Systemic Lupus Erythematosus [20]; LIT – questionnaire inspired by "Lupus Impact Tracker" [13]; MRC – Medical Research Council Dyspnoea Scale; "Dry cough" – participants asked if they had dry cough; "Tightness in chest" – participants were asked if they felt tightness in their chest.

Appendix 2a. Pulmonary function tests of 185 patients with SLE, stratified by presence of pulmonary diseases.

**Pulmonary function test measures.**	**N**	**PD present**	**N**	**PD absent**	**Contrast between groups (95%CI)**
Body plethysmography:					
FEV_1, %* (n = 184)	109	87 ± 18.3	75	99 ± 14.3	13 (7.7 to 18)
FEV_1 low**, no. (%) (n = 184)	109	37 (34%)	75	8 (11%)	23% (10% to 36%)
FVC, %* (n = 184)	109	93 ± 18.4	75	103 ± 14.3	10.0 (5.0 to 15)
FVC low**, no. (%) (n = 184)	109	25 (23%)	75	6 (8.0%)	15% (3.9% to 26%)
FEV_1/FVC (ratio) ^ (n = 184)	109	76 (68 to 80)	75	79 (74 to 81)	3 (0.32 to 5.7)
FEV_1/FVC below 70%, no. (%) (n = 184)	109	28 (26%)	75	10 (13%)	12% (-0.25% to 25%)
TLC, %* (n = 182)	108	96 ± 18.4	74	106 ± 13.8	10 (5.1 to 15.)
TLC low**, no. (%) (n = 182)	108	22 (20%)	74	2 (2.7%)	18% (8.4% to 27%)
Diffusion test:					
DLCO, %* (n = 183)	109	70 ± 16.0	74	81 ± 12.2	11 (6.3 to 15)
DLCO low**, no. (%) (n = 183)	109	79 (72%)	74	34 (46%)	27% (4.3% to 49%)
KCO, %* ^ (n = 183)	109	82 (73 to 92)	74	84 (75 to 94)	2 (-3.3 to 7.3)
KCO low**, no. (%) (n = 183)	109	47 (43%)	74	27 (36%)	6.6% (-12% to 25)
6 min walk test:					
Distance, %* (n = 178)	105	94 ± 17.3	73	95 ± 13.9	0.98 (-3.8 to 5.8)
Distance low**, no. % (n = 178)	105	22 (21%)	73	10 (14%)	7.4% (-4.7% to 20%)
Absolut change in saturation, % ^ (n = 146)	89	3 (2 to 5)	57	2 (1 to 4)	1 (-0.09 to 2.1)
Desaturation below 93% #, no. % (n = 146)	89	15 (17%)	57	6 (11%)	6.3% (-5.7% to 18%)

Value will be means and SDs unless otherwise indicated. * - % of expected; ** - low is defined as below 80% of expected; # - If COPD or emphysema 88%; PD – Pulmonary Diseases; ILD present – ILD according to multidisciplinary discussion; FEV1 – Forced Expiratory Volume in One Second; FVC – Forced Vital Capacity; TLC – Total Lung Capacity; DLCO - Diffusing capacity of the Lung for Carbon Monoxide; KCO – Carbon Monoxide Transfer Coefficient. Contrasts for continuous measures will be based on the difference between means with 95% CIs, while dichotomous outcomes will be based on risk differences with 95% CIs.

Appendix 2b. Pulmonary function tests of 185 patients with SLE, stratified by presence of interstitial lung diseases.

Pulmonary function test measures.	N	ILD present	N	ILD absent	Contrast between groups (95%CI)
Body plethysmography:					
FEV_1, %* (n = 184)	22	78 ± 20	162	94 ± 17	16 (7.8 to 23)
FEV_1 low**, no. (%) (n = 184)	22	10 (45%)	162	35 (22%)	24% (-5.2% to 53%)
FVC, %* (n = 184)	22	84 ± 20	162	99 ± 16	16 (8.0 to 23)
FVC low**, no. (%) (n = 184)	22	7 (32%)	162	24 (15%)	17% (-7.3% to 41%)
FEV_1/FVC (ratio) ^ (n = 184)	22	74 (68 to 80)	162	77 (71 to 81)	3 (-1.0 to 7.0)
FEV_1/FVC below 70%, no. (%)(n = 184)	22	6 (27%)	162	32 (20%)	7.5% (-15% to 30%)
TLC, %* (n = 182)	22	86± 18	160	101 ± 17	15 (7.5 to 23)
TLC low**, no. (%) (n = 182)	22	6 (27%)	160	18 (11%)	16% (-6.4% to 38%)
Diffusion test:					
DLCO, %* (n = 183)	22	62 ± 18	161	76 ± 14	14 (7.1 to 20)
DLCO low**, no. (%) (n = 183)	22	17 (77%)	161	96 (60%)	18% (-21% to 56%)
KCO, %* ^ (n = 183)	22	83 (73 to 91)	161	82 (74 to 93)	2 (-5.7 to 9.7)
KCO low**, no. (%) (n = 183)	22	8 (36%)	161	66 (41%)	4.6% (-22% to 32%)
6 min walk test:					
Distance, %* (n = 178)	20	91 ± 19	158	95 ± 16	4.2 (3.3 to -12)
Distance low**, no. % (n = 178)	20	5 (25%)	158	27 (17%)	7.9% (-15% to 31%)
Desaturation, (Saturation delta) ^ (n = 146)	19	3 (2 to 6)	127	3 (1 to 5)	0 (-1.3 to 1.3)
Desaturation below 93% #, no. % (n = 146)	19	3 (16%)	127	18 (14%)	1.6% (-17% to 21%)

Value will be means and SDs unless otherwise indicated. * - % of expected; ** - low is defined as below 80% of expected; # - If COPD or emphysema 88%; ILD – Interstitial Lung Disease; ILD present – ILD according to multidisciplinary discussion; FEV1 – Forced Expiratory Volume in One Second; FVC – Forced Vital Capacity; TLC – Total Lung Capacity; DLCO - Diffusing capacity of the Lung for Carbon Monoxide; KCO – Carbon Monoxide Transfer Coefficient. Contrasts for continuous measures will be based on the difference between means with 95% CIs, while dichotomous outcomes will be based on risk differences with 95% CIs.

Appendix 2c. Pulmonary function tests of 185 patients with SLE, stratified by presence of pleural diseases.

Pulmonary function test measures.	N	Pleural disease present	N	Pleural disease absent	Contrast between groups (95%CI)
Body plethysmography:					
FEV_1, %*^, (n = 184)	35	84 (71 to 95)	149	97 (82 to 106)	13 (5.1 to 21)
FEV_1 low**, no. (%),(n = 184)	35	13 (37%)	149	32 (21%)	16% (-5.9% to 37%)
FVC, %* ^, (n = 184)	35	91 (79 to 105)	149	101 (90 to 111)	10 (3.0 to 17)
FVC low**, no. (%), (n = 184)	35	9 (26%)	149	22 (14.8%)	11% (-6.9% to 29%)
FEV_1/FVC (ratio) ^, (n = 184)	35	74 (66 to 77)	149	77 (71 to 81)	3 (-0.25 to 6.3)
FEV_1/FVC below 70%, no. (%), (n = 184)	35	11 (31%)	149	27 (18%)	13% (-6.5% to 33%)
TLC, %*^ (n = 182)	35	92 (77 to 98)	147	104 (91 to 113)	12 (4.4 to 20)
TLC low**, no. (%) (n = 182)	35	9 (26%)	147	15 (10%)	16% (-2.1% to 33%)
Diffusion test:					
DLCO, %* (n =183)	35	70 ± 15	148	76 ± 15	5.9 (0.2 to 12)
DLCO low**, no. (%) (n = 183)	35	28 (80%)	148	85 (57%)	23% (-9.5% to 55%)
KCO, %* ^ (n = 183)	35	87 (77 to 97)	148	82 (73 to 93)	5 (-1.2 to 11)
KCO low**, no. (%) (n = 183)	35	11 (31%)	148	63 (43%)	11% (-10% to 32%)
6 min walk test:					
Distance, %* (n = 178)	34	91 ± 16	144	95 ± 16	4.4 (-1.6 to 10)
Distance low**, no. % (n = 178)	34	8 (24%)	144	24 (17%)	6.9% (-11% to 24%)
Desaturation, (Saturation delta)^ (n = 146)	33	2 (1 to 4)	113	3 (1 to 5)	1 (0.06 to 1.9)
Desaturation below 93% #, no. % (n = 146)	33	4 (12%)	113	17 (15%)	2.9% (-11% to 17%)

Value will be means and SDs unless otherwise indicated. * - % of expected; ** - low is defined as below 80% of expected; # - If COPD or emphysema 88%; Pleural disease present – Pleural Disease according to multidisciplinary discussion; FEV1 – Forced Expiratory Volume in One Second; FVC – Forced Vital Capacity; TLC – Total Lung Capacity; DLCO - Diffusing capacity of the Lung for Carbon Monoxide; KCO – Carbon Monoxide Transfer Coefficient. Contrasts for continuous measures will be based on the difference between means with 95% CIs, while dichotomous outcomes will be based on risk differences with 95% CIs.

Appendix 2d. Pulmonary function tests of 185 patients with SLE, stratified by presence of airway diseases.

Pulmonary function test measures.	N	Airway disease present	N	Airway disease absent	Contrast between groups (95%CI)
Body plethysmography:					
FEV_1, %*, (n = 184)	70	88 ± 19	114	94 ± 17	6.7 (1.4 to -12)
FEV_1 low**, (n = 184)	70	24 (34%)	114	21 (18%)	16% (0.05% to 32%)
FVC, %* ^, (n = 184)	70	98 (85 to 111)	114	101 (89 to 109)	3 (-2.9 to 8.9)
FVC low**, no. (%),(n = 184)	70	16 (23%)	114	15 (13%)	9.7% (-3.3% to 23%)
FEV_1/FVC (ratio) ^, (n = 184)	70	75 (67 to 80)	114	77 (74 to 81)	3 (0.31 to 5.7)
FEV_1/FVC below 70%, no. (%), (n = 184)	70	23 (33%)	114	15 (13%)	20% (4.7% to 35%)
TLC, %*^ (n = 182)	69	100 (86 to 110)	113	102 (90 to 112)	2 (-5.2 to 9.2)
TLC low**, no. (%) (n = 182)	69	15 (22%)	113	9 (8.0%)	14% (1.6% to 26%)
Diffusion test:					
DLCO, %* (n = 183)	70	71 ± 16	113	76 ± 15	5.0 (-0.45 to 9.6)
DLCO low**, no. (%) (n = 183)	70	49 (70%)	113	64 (57%)	13% (-11% to 37%)
KCO, %* ^ (n = 183)	70	82 (70 to 91)	113	82 (75 to 93)	0 (-4.9 to 4.9)
KCO low**, no. (%) (n = 183)	70	33 (47%)	113	41 (36%)	11% (-8.7% to 30%)
6 min walk test:					
Distance, %* (n = 178)	69	97 ± 17	109	92 ± 15	4.4 (-0.46 to 9.2)
Distance low**, no. % (n = 178)	69	11 (16%)	109	21 (19%)	3.3 (-9.2 to 16)
Desaturation, (Saturation delta) (n = 146)	53	3 (2 to 6)	93	2 (1 to 4)	0 (-1.4 to 1.4)
Desaturation below 93% #, no. % (n = 146)	53	8 (15%)	93	13 (14%)	1.1% (-12% to 14%)

Value will be means and SDs unless otherwise indicated. *- % of expected; ** - low is defined as below 80% of expected; # - If COPD or emphysema 88%; Airway disease positive – Airway Disease according to multidisciplinary discussion; FEV1 – Forced Expiratory Volume in One Second; FVC – Forced Vital Capacity; TLC – Total Lung Capacity; DLCO - Diffusing capacity of the Lung for Carbon Monoxide; KCO – Carbon Monoxide Transfer Coefficient. Contrasts for continuous measures will be based on the difference between means with 95% CIs, while dichotomous outcomes will be based on risk differences with 95% CIs.

Appendix 3. The prevalence of High-Resolution CT findings among 185 patients with SLE.

Groups of HRCT findings	Subgroups of HRCT findings	Positive, no. (%)	95% CI
Any abnormality		107 (58%)	51% to 65%
Interstitial lung disease		22 (12%)	7.9% to 17%
	Definite UIP	2 (1.1%)	0.027% to 4.3%
	Probable UIP	5 (2.7%)	1.1% to 6.4%
	Indeterminate for UIP	1 (0.54%)	0.0075% to 3.8%
	NSIP fibrotic	3 (1.6%)	0.52% to 4.9%
	NSIP non-fibrotic	2 (1.1%)	0.027% to 4.3%
	HP fibrotic	1 (0.54%)	0.0075% to 3.8%
	HP non-fibrotic	2 (1.1%)	0.027% to 4.3%
	LIP	1 (0.54%)	0.0075% to 3.8%
	OP fibrotic	0	NA
	OP non-fibrotic	0	NA
	ILA	6 (3.2%)	1.5% to 7.1%
	Other	1 (0.54%)	0.0075% to 3.8%
Airways		46 (25%)	19% to 32%
	Bronchiectasis	40 (22%)	16% to 28%
	Bronchial wall thickening	10 (5.4%)	2.9% to 9.8%
	Bronchiolitis	4 (2.2%)	0.81% to 5.6%
	Air trapping	12 (6.5%)	3.7% to 11%
	Other	0	NA
Pleural		46 (25%)	19% to 32%
	Pleural thickening	46 (25%)	19% to 32%
	Pleural effusion	2 (1.1%)	0.027% to 4.3%
	Pleuroparenchymal fibrosis	16 (8.6%)	5.3% to 14%
	Other	5 (2.7%)	1.1% to 6.4%
Elevated diaphragm		16 (8.7%)	5.3% to 14%
Suspect of malignancy		6 (3.2%)	1.5% to 7.1%
Emphysema		17 (9.2%)	5.8% to 14%
Nodules		22 (12%)	7.9% to 17%
Cysts		16 (8.7%)	5.3% to 14%
Enlarged pulmonary arteries		13 (7.0%)	4.1% to 12%
Other		3 (1.6%)	0.52% to 4.9%

Percentage is of the total scans. HRCT – High-Resolution Computed Tomography scan; 95% Confidence interval of prevalence; NA – Not applicable; UIP – Usual Interstitial Pneumonia; NSIP – Non-Specific Interstitial Pneumonia; HP – Hypersensitivity Pneumonitis; LIP – Lymphoid Interstitial Pneumonia; OP – Organising Pneumonia; ILA – Interstitail lung abnormalities.

Appendix 4. Impact of baseline characteristics and patient related outcome measures that are not presented in table 3 in SLE, according to groups of pulmonary diseases investigated by univariable logistic regression analyses.

**Outcome measure / Baseline characteristic**	**PD OR (95%CI)**	**ILD OR (95%CI)**	**Pleural dis OR (95%CI)**	**Airway dis OR (95%CI)**
White population no. n = 173/185 (94%)	2.1 (0.64 to 6.9)	1.5 (0.19 to 12)	2.7 (0.34 to 22)	0.84 (0.26 to 2.8)
BMI, kg/m^2	1.01 (0.96 to 1.1)	1.03 (0.95 to 1.1)	1.0 (0.95 to 1.1)	0.99 (0.94 to 1.05)
Overlap to any ARD, n = 88/185 (48%)	1.5 (0.81 to 2.6)	2.6 (1.02 to 6.8)	2.2 (1.01 to 4.6)	0.89 (0.49 to 1.6)
Antiphospholipid Syndrome, n = 42/185 (23%)	2.0 (0.96 to 4.3)	2.4 (0.93 to 6.3)	4.4 (2.0 to 9.9)	0.89 (0.44 to 1.8)
Sjogren's Syndrome, n =25/185 (14%)	1.1 (0.45 to 2.5)	2.8 (0.99 to 8.2)	1.4 (0.52 to 3.9)	0.74 (0.30 to 1.8)
Rheumatoid Arthritis, n = 18/185 (10%)	1.4 (0.52 to 4.0)	0.92 (0.20 to 4.3)	0.23 (0.03 to 1.8)	1.4 (0.51 to 3.6)
Systemic Sclerosis, n = 3/185 (1.6%)	0.34 (0.03 to 3.9)	No SSc with ILD	2.2 (0.19 to 25)	No SSc with airway dis
Former, Mixed Connective Tissue Disease, n = 10/185 (5.4%)	1.1 (0.29 to 3.9)	0.82 (0.10 to 6.8)	1.1 (0.22 to 5.3)	1.7 (0.47 to 6.1)
Pleural effusion, n = 42/185 (23%)	/	2.2 (0.84 to 5.6)	/	1.0 (0.50 to 2.1)
Positive aPL ever, n = 86/185 (46%)	2.1 (1.2 to 3.9)	1.8 (0.72 to 4.4)	2.0 (0.93 to 4.2)	1.3 (0.69 to 2.3)
Decreased complement ever, n = 138/185 (75%)	0.96 (0.49 to 1.9)	1.2 (0.41 to 3.4)	1.8 (0.70 to 4.7)	0.61 (0.31 to 1.2)
Treatment:				
Corticosteroid ever, 167/185 (90%)	2.5 (0.91 to 6.7)	All ILD = corticosteroid ever	2.0 (0.43 to 9.0)	1.2 (0.44 to 3.5)
Corticosteroid current, 49/185 (26%)	1.6 (0.82 to 3.2)	0.80 (0.28 to 2.3)	1.1 (0.50 to 2.6)	0.94 (0.48 to 1.8)
Hydroxychloroquine ever, 185/185 (100%)	All had received hydroxychloro quin	All had received hydroxychloro quin	All had received hydroxychloro quin	All had received hydroxychloro quin
Hydroxychloroquine current, 143/185 (77%)	0.57	0.46	0.57	0.52

	(0.27 to 1.2)	(0.18 to 1.2)	(0.25 to 1.3)	(0.26 to 1.05)
Other csDMARDs ever, 154/185 (83%)	1.04<br>(0.48 to 2.3)	2.2<br>(0.48 to 9,8)	1.7<br>(0.55 to 5.2)	0.81<br>(0.37 to 1.8)
Other csDMARDs current, 83/185 (45%)	0.84<br>(0.47 to 1.5)	1.03<br>(0.42 to 2.5)	0.58<br>(0.27 to 1.3)	0.66<br>(0.36 to 1.2)
BioDMARDs ever, 36/185 (19%)	1.3<br>(0.61 to 2.8)	0.62<br>(0.17 to 2.2)	1.3<br>(0.53 to 3.1)	0.78<br>(0.36 to 1.7)
BioDMARDs current, 17/185 (9.2%)	1.8<br>(0.59 to 5.2)	0.44<br>(0.06 to 3.5)	0.55<br>(0.12 to 2.5)	2.0<br>(0.72 to 5.4)
SLE specific disease scores:				
Classification criteria EULAR/ACR criteria, score 0-51	1.04<br>(1.00 to 1.09	1.00<br>(0.94 to 1.06)	1.05<br>(0.997 to 1.1)	1.02<br>(0.98 to 1.06)
SLEDAI 2K, without PD, score 0-103	0.91<br>(0.83 to 1.0)	0.84<br>(0.70 to 1.0)	1.0<br>(0.90 to 1.1)	0.96<br>(0.87 to 1.1)
SDI, without PD, score 0-43	1.3<br>(1.1 to 1.6)	1.3<br>(1.1 to 1.5)	1.2<br>(1.0 to 1.4)	1.2<br>(1.0 to 1.4
PGA, score 0-3	0.82<br>(0.39 to 1.7)	0.83<br>(0.26 to 2.6)	1.1<br>(0.45 to 2.9)	0.89<br>(0.42 to 1.9)
LLDAS achieved, 124/185 (67%)	1.2<br>(0.65 to 2.3)	1.4<br>(0.50 to 3.7)	0.93<br>(0.43 to 2.0)	1.1<br>(0.59 to 2.1)
DORIS remission achieved 69/185 (37%)	1.03<br>(0.56 to 1.9)	0.96<br>(0.38 to 2.4)	0.85<br>(0.39 to 1.8)	1.3<br>(0.72 to 2.4)
PROMs:				
LIT, score 0-100	1.0<br>(0.98 to 1.01)	1.0<br>(0.98 to 1.03)	1.0<br>(0.98 to 1.02)	1.00<br>(0.99 to 1.02)
SLAQ, score 0-100	1.0<br>(0.97 to 1.03)	1.0<br>(0.95 to 1.05)	0.99<br>(0.96 to 1.03)	1.01<br>(0.98 to 1.05)
MRC, score 1-5<br>1<br>2<br><br>3<br><br>4<br><br>5	 Ref<br>1.7<br>(0.86 to 3.3)<br>3.1<br>(1.06 to 9.2)<br>All PD<br><br>Empty	 Ref<br>1.9<br>(0.66 to 5.3)<br>1.9<br>(0.46 to 7.8)<br>17<br>(2.5 to 118)<br>Empty	 Ref<br>1.03<br>(0.44 to 2.4)<br>2.4<br>(0.85 to 6.9)<br>1.2<br>(0.13 to 12)<br>Empty	 Ref<br>1.2<br>(0.59 to 2.3)<br>2.7<br>(1.02 to 7.0)<br>1.3<br>(0.21 to 8.4)<br>Empty
Dry cough, status, n = 182<br>Never, n = 123 (68%)	0.36*<br>0.76<br>(0.40 to 1.4)	0.66*<br>0.66<br>(0.26 to 1.6)	0.35*<br>0.57<br>(0.27 to 1.2)	0.84*<br>1.2<br>(0.63 to 2.3)

Prior, n = 12 (6.6%)	0.65<br>(0.20 to 2.1)	1.5<br>(0.31 to 7.3)	1.4<br>(0.37 to 5.6)	0.79<br>(0.23 to 2.7)
Present, n = 47 (26%)	1.6<br>(0.80 to 3.2)	1.4<br>(0.53 to 3.7)	1.7<br>(0.75 to 3.7)	0.88<br>(0.44 to 1.7)
Tightness in chest, status, n = 182	0.32*	0.02*	0.15*	0.96*
Never, n = 100 (55%)	0.84<br>(0.46 to 1.5)	0.42<br>(0.17 to 1.1)	0.55<br>(0.26 to 1.2)	1.1<br>(0.58 to 1.9
Prior, n = 17 (9.3%)	2.3<br>(0.73 to 7.5)	5.1<br>(1.7 to 17)	2.6<br>(0.88 to 7.5)	0.86<br>(0.30 to 2.4)
Present, n = 65 (36%)	0.91<br>(0.49 to 1.7)	1.0<br>(0.41 to 2.6)	1.3<br>(0.59 to 2.7)	1.0<br>(0.54 to 1.9)
Body plethysmography:				
FEV_1, %	0.95<br>(0.94 to 0.97)	0.95<br>(0.93 to 0.98)	0.97<br>(0.95 to 0.99)	0.98<br>(0.96 to 0.996)
FEV_1 low~, 45/184 (24%)	4.3<br>(1.9 to 9.9)	3.0<br>(1.2 to 7.6)	2.2<br>(0.98 to 4.8)	2.3<br>(1.2 to 4.6)
FVC, %	0.96<br>(0.94 to 0.98)	0.95<br>(0.93 to 0.98)	0.98<br>(0.96 to 0.995)	0.99<br>(0.97 to 1.0)
FVC low~, 31/184 (17%)	3.4<br>(1.3 to 8.8)	2.7<br>(0.99 to 7.3)	2.0<br>(0.83 to 4.8)	2.0<br>(0.90 to 4.3)
FEV_1/FVC (ratio)	0.94<br>(0.91 to 0.98)	0.97<br>(0.92 to 1.02)	0.96<br>(0.91 to 0.999)	0.94<br>(0.90 to 0.98)
FEV_1/FVC below 70%, 38/184 (21%)	2.3<br>(1.02 to 5.0)	1.5<br>(0.55 to 4.2)	2.1<br>(0.91 to 4.7)	3.2<br>(1.6 to 6.8)
TLC, %	0.96<br>(0.94 to 0.98)	0.95<br>(0.93 0.98)	0.96<br>(0.94 to 0.98)	0.99<br>(0.98 to 1.01)
TLC low~, 24/182 (13%)	9.2<br>(2.1 to 41)	3.0<br>(1.03 to 8.5)	3.1<br>(1.2 to 7.7)	3.2<br>(1.3 to 7.8)
Diffusion test:				
DLCO, %	0.95<br>(0.93 to 0.97)	0.94<br>(0.91 to 0.97)	0.98<br>(0.95 to 0.999)	0.98<br>(0.96 to 0.998)
DLCO low~, 113/183 (62%)	3.1<br>(1.7 to 5.8)	2.3<br>(0.81 to 6.6)	3.0<br>(1.2 to 7.2)	1.8<br>(0.95 to 3.4)
KCO, %	0.99<br>(0.97 to 1.01)	0.999<br>(0.97 to 1.0)	1.02<br>(0.99 to 1.04)	0.99<br>(0.97 to 1.01)
KCO low~, 74/183 (40%)	1.3<br>(0.72 to 2.4)	0.82<br>(0.33 to 2.1)	0.62<br>(0.28 to 1.4)	1.6<br>(0.85 to 2.9)
6 min walk test:				
Distance, %	0.996	0.98	0.98	1.02

	(0.98 to 1.01)	(0.96 to 1.01)	(0.96 to 1.01)	(0.998 to 1.04)
Distance low˜, 32/178 (18%)	1.7 (0.75 to 3.8)	1.6 (0.55 to 4.9)	1.6 (0.63 to 3.8)	0.80 (0.36 to 1.8)
Desaturation (Saturation delta)	0.98 (0.91 to 1.1)	1.03 (0.91 to 1.2)	0.96 (0.86 to 1.1)	0.96 (0.88 to 1.04)
Desaturation below 93% #, 21/146 (14%)	1.3 (0.51 to 3.4)	2.7 (0.76 to 9.6)	1.04 (0.32 to 3.4)	0.49 (0.17 to 1.4)

Table of planned univariable analysis. *According to EULAR/ACR 2019 criteria [12]. Dis - Disease ˜ - low is defined as below 80% of expected; ^ - % of expected; # - If COPD or emphysema 88%. PD – Pulmonary according to multidisciplinary discussion; OR - Odds ration; 95% CI – 95% Confidence Interval; Airway – Airway Disease according to multidisciplinary discussion; Pleural – Pleural Disease according to multidisciplinary discussion; ILD – Interstitial Lung Disease positive according to multidisciplinary discussion; SLS – Shrinking Lung Syndrome according to multidisciplinary discussion; BMI – Body Mass Index; ARD – Autoimmune Rheumatic Diseases; Pleural effusion – defined as pleural effusion ever according to medical record; aPL– Antiphospholipid Antibodies; csDMARD – Conventional Disease-Modifying Antirheumatic Drugs; bioDMARDs – Biologic Disease-Modifying Antirheumatic Drugs; SLEDAI 2K – Systemic Lupus Erythematosus Disease Activity Index 2000 [16]; PGA – Physician Global Assessment [17]; LLDAS – Lupus Low Disease Activity State [18]; DORIS – Definition of Remission in SLE [19]; SDI – Systemic Lupus International Collaborating Clinics /American College of Rheumatology Damage Index for Systemic Lupus Erythematosus; PROMs – Patient Reported Outcome Measures; LIT – questionnaire inspired by “Lupus Impact Tracker” [13]; SLAQ – Systemic Lupus Activity Questionnaire [29]; Ref – Reference; MRC – Medical Research Council Dyspnoea Scale; FEV1 – Forced Expiratory Volume in One Second; FVC – Forced Vital Capacity; TLC – Total Lung Capacity; DLCO - Diffusing capacity of the Lung for Carbon Monoxide; KCO – Carbon Monoxide Transfer Coefficient.

Appendix 5. Test characteristics of pulmonary function tests in 185 patients with SLE, divided by presence of pulmonary diseases and subtypes of pulmonary diseases.

Measurement	Sensitivity	Specificity	BA	PPV	NPV	Positive rate
Pulmonary diseases						
FEV1	34%	89%	62%	82%	48%	24%
FVC	23%	92%	58%	81%	45%	17%
FEV1/FVC	26%	87%	57%	74%	45%	21%
TLC	20%	97%	59%	92%	46%	13%
DLCO	72%	54%	63%	70%	57%	62%
KCO	43%	64%	54%	64%	43%	40%
Distance	21%	86%	54%	69%	44%	18%
Sat low	17%	89%	53%	71%	41%	14%
Interstitial lung diseases						
FEV1	45%	78%	62%	22%	91%	24%
FVC	32%	85%	59%	23%	90%	17%
FEV1/FVC	27%	80%	54%	21%	89%	21%
TLC	27%	89%	58%	25%	90%	13%
DLCO	77%	40%	59%	15%	93%	62%
KCO	36%	59%	48%	11%	87%	40%
Distance	25%	83%	54%	16%	90%	18%
Sat low	16%	86%	51%	14%	87%	14%
Pleural diseases						
FEV1	37%	79%	58%	29%	84%	24%
FVC	26%	85%	56%	29%	83%	17%
FEV1/FVC	31%	82%	57%	29%	84%	21%
TLC	26%	90%	58%	38%	84%	13%
DLCO	80%	43%	62%	25%	90%	62%
KCO	31%	57%	44%	15%	78%	40%
Distance	24%	83%	54%	25%	82%	18%
Sat low	12%	85%	49%	19%	77%	14%
Airway diseases						
FEV1	34%	82%	58%	53%	67%	24%
FVC	23%	87%	55%	52%	65%	17%
FEV1/FVC	33%	87%	60%	61%	68%	21%

TLC	22%	92%	57%	63%	66%	13%
DLCO	70%	43%	57%	43%	70%	62%
KCO	47%	64%	56%	45%	66%	40%
Distance	16%	81%	49%	34%	60%	18%
Sat low	15%	86%	51%	38%	64%	14%

BA - balanced accuracy, PPV – positive predictive value, NPV – negative predictive value, FEV1 – forced expiratory volume in one second, FVC – forced vital capacity, TLC – total lung capacity, DLCO - Diffusing Capacity for the Lung for Carbon Monoxide, KCO - Carbon Monoxide Transfer Coefficient.

Appendix 6 a. Analyses of predictor variables, whose 95% confidence interval for odds ratio did not include one regarding the association to any pulmonary diseases in 185 patients with SLE.

**Predictor**	**Univariable analyses, OR (95%CI)**	**Multivariable analysis, OR (95%CI)**	**Fitted analysis, OR (95%CI)**
Age, years	1.05 (1.03 to 1.08)	1.08 (1.02 to 1.14)	1.05 (1.02 to 1.08)
Disease duration, years	0.97 (0.95 to 1.0)	1.00 (0.96 to 1.04)	NA
SDI, Score 0-48	1.5 (1.2 to 1.8)	11 (3.1 to 37)	1.2 (0.96 to 1.4)
SDI no PD, Score 0-43	1.3 (1.1 to 1.6)	0.09 (0.03 to 0.32)	Omitted
Positive aPL ever	2.1 (1.2 to 3.9)	2.2 (0.95 to 5.2)	2.2 (1.1 to 4.4)
EULAR/ACR 2019, score 0-51 [12]	1.04 (1.00 to 1.09)	1.03 (0.97 to 1.1)	NA
MRC, score 3	3.1 (1.06 to 9.2)	1.5 (0.40 to 5.9)	NA
FEV_1, %	0.95 (0.94 to 0.97)	0.89 (0.75 to 1.1)	NA
FEV_1 low	4.3 (1.9 to 9.9)	1.2 (0.19 to 7.5)	NA
FVC, %	0.96 (0.99 to 0.98)	1.1 (0.91 to 1.3)	NA
FVC low	3.4 (1.3 to 8.8)	0.25 (0.03 to 2.2)	1.1 (0.26 to 4.6)
FEV_1/FVC (ratio)	0.94 (0.91 to 0.98)	1.1 (0.91 to 1.4)	NA
FEV_1/FVC below 70%	2.3 (1.02 to 5.0)	0.64 (0.12 to 3.4)	NA
TLC, %	0.96 (0.94 to 0.98)	1.02 (0.97 to 1.1)	NA
TLC low	9.21 (2.09 to 40.5)	3.26 (0.372 to 28.5)	5.14 (0.747 to 35.4)
DLCO, %	0.95 (0.93 to 0.97)	0.99 (0.94 to 1.03)	0.97(0.94 to 0.995)
DLCO low	3.1 (1.7 to 5.8)	1.4 (0.40 to 5.0)	NA

OR – Odds ratio, 95%CI – 95% Confidence Interval; NA – Not Applicable; SDI – Systemic Lupus International Collaborating Clinics /American College of Rheumatology Damage Index for Systemic Lupus Erythematosus, PD – Pulmonary Diseases, aPL – anti-Phospholipid antigen; MRC – Medical Research Counsil dyspnoea scale, FEV_1 – Forced Expiratory Volume in one second; FVC – Forced Vital Capacity; TLC – Total Lung Capacity; DLCO – Diffusing capacity of the Lung for Carbon Monoxide. % - Percentage of what is expected according to age, hight, weight, and sex, low – below 80% of what is expected.

Appendix 6 b. Analyses of predictors that were significantly associated to interstitial lung in 185 patients with diseases in SLE.

Predictor	Univariable analyses, OR (95%CI)	Multivariable analysis, OR (95%CI)	Fitted analysis, OR (95%CI)
Age, years	1.06 (1.03 to 1.1)	1.06 (1.01 to 1.1)	1.06 (1.02 to 1.1)
SDI, Score 0-48	1.4 (1.2 to 1.7)	8.6 (2.6 to 29)	1.4 (1.1 to 1.7)
SDI no pd, Score 0-43	1.3 (1.05 to 1.5)	0.14 (0.04 to 0.47)	NA
Pleuritis positive	3.4 (1.2 to 9.5)	0.75 (0.16 to 3.5)	NA
"Tightness in chest", prior	5.1 (1.7 to 16)	1.3 (0.16 to 9.8)	NA
Overlap to other ARD	2.6 (1.02 to 6.8)	1.02 (0.26 to 4.0)	NA
MRC, score 4	17 (2.5 to 118)	1.5 (0.09 to 24)	NA
FEV_1, %	0.95 (0.93 to 0.98)	1.03 (0.94 to 1.1)	NA
FEV_1 low	3.0 (1.2 to 7.6)	0.44 (0.05 to 3.8)	0.32 (0.07 to 1.6)
FVC, %	0.95 (0.93 to 0.98)	0.94 (0.85 to 1.0)	0.91 (0.87 to 0.96)
TLC, %	0.95 (0.93 to 0.98)	0.96 (0.88 to 1.04)	NA
TLC low	3.0 (1.03 to 8.5)	0.11 (0.009 to 1.5)	0.40 (0.08 to 2.0)
DLCO, %	0.94 (0.91 to 0.97)	0.98 (0.93 to 1.04)	NA

OR – Odds ratio; 95%CI – 95% Confidence Interval; SDI – Systemic Lupus International Collaborating Clinics /American College of Rheumatology Damage Index for Systemic Lupus Erythematosus; PD – Pulmonary Diseases; NA – Not Applicable; Pleuritis positive – Pleuritis according to definition in SLICC 2012 [28]; ARD – Autoimmune Rheumatic Diseases; MRC – Medical Research Counsil dyspnoea scale, FEV_1 – Forced Expiratory Volume in one second; FVC – Forced Vital Capacity; TLC – Total Lung Capacity; DLCO – Diffusing capacity of the Lung for Carbon Monoxide. % - Percentage of what is expected according to age, hight, weight, and sex, low – below 80% of what is expected.

Appendix 6 c. Analyses of predictors that were significantly associated to pleural diseases in 185 patients with SLE.

Predictor	Univariable analyses, OR (95%CI)	Multivariable analysis, OR (95%CI)	Fitted analysis, OR (95%CI)
SDI, Score 0-48	1.4 (1.2 to 1.6)	577 (13 to 24)	1.2 (1.04 to 1.5)
SDI no pd, Score 0-43	1.2 (1.03 to 1.4)	0.017 (0.004 to 0.08)	NA
Overlap to other ARD	2.2 (1.01 to 4.6)	0.75 (0.16 to 3.6)	NA
APS	4.4 (2.0 to 9.9)	7.0 (1.2 to 41)	2.5 (1.03 to 6.1)
FEV_1, %	0.97 (0.95 to 0.99)	0.94 (0.80 to 1.1)	0.98 (0.96 to 1.0)
FVC, %	0.98 (0.96 to 0.995)	1.09 (0.92 to 1.3)	NA
FEV_1/FVC (ratio)	0.96 (0.91 to 0.999)	1.0 (0.83 to 1.2)	NA
TLC, %	0.96 (0.94 to 0.99)	0.97 (0.90 to 1.04)	NA
TLC low	3.1 (1.2 to 7.7)	0.98 (0.12 to 8.0)	NA
DLCO, %	0.98 (0.95 to 0.999)	1.1 (1.04 to 1.2)	NA
DLCO low	3.0 (1.2 to 7.2)	27 (3.0 to 240)	NA

OR – Odds ratio; 95%CI – 95% confidence interval; SDI – Systemic Lupus International Collaborating Clinics /American College of Rheumatology Damage Index for Systemic Lupus Erythematosus; PD – Pulmonary Diseases; NA – Not Applicable; ARD – Autoimmune Rheumatic Diseaes; APS – anti-Phospholipid Syndrome; FEV_1 – Forced Expiratory Volume in one second; FVC – Forced Vital Capacity; TLC – Total Lung Capacity; DLCO – Diffusing capacity of the Lung for Carbon Monoxide. % - Percentage of what is expected according to age, hight, weight, and sex, low – below 80% of what is expected.

Appendix 6 d. Analyses of predictors that were significantly associated to airway diseases in 185 patients with SLE.

**Predictor**	**Univariable analyses, OR (95%CI)**	**Multivariable analysis, OR (95%CI)**	**Fitted analysis, OR (95%CI)**
Age, years	1.03 (1.01 to 1.06)	1.02 (0.995 to 1.05)	1.02 (0.998 to 1.05)
SDI, Score 0-48	1.2 (1.02 to 1.3)	0.82 (0.44 to 1.5)	NA
SDI no pd, Score 0-43	1.2 (1.01 to 1.4)	1.3 (0.67 to 2.5)	NA
MRC score 3	2.7 (1.02 to 7.0)	1.7 (0.57 to 4.7)	NA
FEV_1, %	0.98 (0.96 to 0.996)	1.0 (0.97 to 1.04)	NA
FEV_1 low	2.3 (1.2 to 4.6)	1.2 (0.36 to 4.3)	NA
FEV_1/FVC (ratio)	0.94 (0.90 to 0.98)	0.99 (0.92 to 1.07)	NA
FEV_1/FVC below 70%	3.2 (1.6 to 6.8)	2.3 (0.67 to 7.8)	2.8 (1.3 to 6.2)
TLC low	3.2 (1.3 to 7.8)	3.6 (1.02 to 13)	3.7 (1.5 to 9.4)
DLCO %	0.98 (0.96 to 0.998)	0.995 (0.97 to 1.02)	NA

OR – Odds ratio; 95%CI – 95% confidence interval; SDI – Systemic Lupus International Collaborating Clinics /American College of Rheumatology Damage Index for Systemic Lupus Erythematosus; NA – Not Applicable; PD – Pulmonary Diseases; FEV_1 – Forced Expiratory Volume in one second; FVC – Forced Vital Capacity; TLC – Total Lung Capacity; DLCO – Diffusing capacity of the Lung for Carbon Monoxide. % - Percentage of what is expected according to age, hight, weight, and sex, low – below 80% of what is expected.
